# CircNPHP4 in monocyte-derived small extracellular vesicles controls heterogeneous adhesion in coronary heart atherosclerotic disease

**DOI:** 10.1038/s41419-021-04253-y

**Published:** 2021-10-14

**Authors:** Feng Xiong, Rui Mao, Lijuan Zhang, Ruohan Zhao, Kunyue Tan, Chunxia Liu, JunBo Xu, Guanghong Du, Tongtong Zhang

**Affiliations:** 1 Department of Cardiology, Cadiovascular Institute of Chengdu, Chengdu Third People’s hospital, Chengdu, 610031 China; 2 Department of Dermatology, Xiangya Hospital, Central South University, Changsha, China; 3 Geriatric Department, Sichuan Academy of Medical Sciences & Sichuan Provincial People’s Hospital, Chengdu, Sichuan 610072 China; 4 Medical Research Center, The Third People’s Hospital of Chengdu, The Affiliated Hospital of Southwest Jiaotong University, The Second Chengdu Hospital Affiliated to Chongqing Medical University, Chengdu, Sichuan 610031 China; 5 Center of Gastrointestinal and Minimally Invasive Surgery, Department of General Surgery, The Third People’s Hospital of Chengdu, Affiliated Hospital of Southwest Jiaotong University & The Second Affiliated Hospital of Chengdu, Chongqing Medical University, Chengdu, 610031 China

**Keywords:** Non-coding RNAs, Predictive markers, Atherosclerosis

## Abstract

Small extracellular vesicles (sEVs)-derived circular RNAs (circRNAs) could regulate gene expression in recipient cells, and dysregulation of sEVs-derived circRNAs has been implicated in several diseases. However, the expression and function of sEVs-derived circRNAs in coronary heart atherosclerotic disease (CAD) remain unknown. In this study, we investigated global changes in the expression patterns of circRNAs in sEVs from CAD-related monocytes and identified circNPHP4 as a significantly upregulated circRNA. Knockdown of circNPHP4 inhibited heterogeneous adhesion between monocytes and coronary artery endothelial cells and reduced ICAM-1 and VCAM-1 expression. Investigations of the underlying mechanisms revealed that circNPHP4 contains a functional miR-1231-binding site. Mutation of the circNPHP4-binding sites in miR-1231 abolished the interaction, as indicated by a luciferase reporter assay. Furthermore, circNPHP4 affected the expression of miR-1231 and its target gene EGFR. Overexpression of miR-1231 blocked the inhibitory effect of circNPHP4 on heterogeneous adhesion. Moreover, downregulation of miR-1231 restored heterogeneous adhesion upon inhibition by circNPHP4 silencing. Additionally, circNPHP4 overexpression was correlated with aggressive clinicopathological characteristics in CAD patients. A multivariate logistic regression model and bootstrapping validation showed that circNPHP4 overexpression had a good risk prediction capability for CAD. The decision curve analysis revealed that using the CAD nomogram that included circNPHP4 overexpression to predict the risk of CAD was beneficial. Our results suggest that sEVs-derived circNPHP4 can serve as a potential target for CAD treatments or as a potential diagnostic marker for CAD patients.

## Introduction

As one of the most common forms of cardiovascular diseases, CAD remains the main cause of death in Western and developing countries [1, 2]. The underlying pathological process is arterial wall thickening resulting from coronary atherosclerotic plaque formation [3, 4] and can lead to acute coronary syndrome or stroke. Therefore, it is urgent to search for sensitive and specific biomarkers and targeting molecules for the diagnosis and treatment of CAD.

Circular RNAs (circRNAs) have been implicated in gene regulation in a wide variety of organisms [5, 6]. However, the mechanisms by which circRNAs function during disease progression have not been elucidated. It has been suggested that circRNAs may regulate gene expression via different targets in different types of diseases or even at different disease stages [7–9]. Furthermore, emerging evidence has suggested that some circRNAs act as miRNA sponges by modulating gene transcription and interacting with RNA-binding proteins (RBPs) in various diseases, including cancer, obesity, and central nervous system diseases [10–12]. Moreover, several circRNAs have been reported to play an important role in the proliferation, migration, and tube formation of endothelial cells in atherosclerosis [13–15].

sEVs are 40–150 nm nanovesicles released into the extracellular environment via the endosomal vesicle pathway by fusion with the plasma membrane [16]. A broad range of cells, including tumor cells, epithelial cells, and immune cells, secrete sEVs [17], which are essential for intercellular communication [18]. sEVs contain a wide range of contents, including a variety of circRNAs [19], which have been reported to play a major role in the pathophysiological processes of cardiovascular disease [20, 21]. However, no study has reported the role of circRNA in sEVs in CAD.

Monocytes undertake important functions in the pathological process of CAD [22, 23]. Human monocytes have been classified into the CD14^++^CD16^−^ group, CD14^++^CD16^+^ group, and CD14^+^CD16^++^ group [24]. Adhesion of CD14^++^CD16^+^ and CD14^+^CD16^++^ monocytes to the endothelium is important for vascular inflammatory diseases, including CAD [25]. A previous study showed that sEVs from monocytes could play an important role in the heterogeneous adhesion between monocytes and endothelial cells [26–28]. However, to date, no one has studied the role of circRNA in sEVs from monocytes in heterogeneous adhesion between monocytes and endothelial cells in CAD.

In this study, circRNA expression profiles were constructed for monocyte-derived sEVs from CAD patients and control individuals using circRNA microarrays. In CAD individuals, circNPHP4 (hsa_circ_0009135) was significantly upregulated and was related to the severity degree. Functional assays indicated that knockdown of exosomal circNPHP4 inhibited heterogeneous adhesion between monocytes and endothelial cells. Furthermore, circNPHP4 expression was positively correlated with NPHP4 expression in monocytes from CAD patients. The results presented herein show that circNPHP4 binds to miR-1231 and acts as a miRNA sponge to subsequently regulate EGFR expression. Overall, these findings suggest that exosomal circNPHP4 also acts as a heterogeneous adhesion-promoting factor and can serve as a diagnostic biomarker for CAD.

## Materials and methods

### Clinical samples

A total of 109 patients with CAD confirmed by coronary angiography in the Third People’s Hospital of Chengdu, China, between March 2019 and December 2019 were included. In addition, 70 healthy volunteers were enrolled in the control group (Supplementary Table 1). The study was approved by the ethics committee of Chengdu Third People’s Hospital (Chengdu, China; approval no. 2019S-93). And all participants provided written informed consent prior to their enrollment in the study. The inclusion criterion for the CAD group was coronary angiography showing ≥50% stenosis in ≥1 blood vessel. In addition, patients with any tumors, trauma, serious infection, serious liver or kidney disease, or cerebrovascular accident, and pregnant women were excluded.

### Monocyte isolation

Peripheral blood mononuclear cells (PBMCs) from CAD patients and control individuals were isolated by Ficoll-Paque PLUS density gradient centrifugation (GE Healthcare Bioscience). The PBMCs were cryopreserved for less than 3 months at −80 °C in 90% heat-inactivated fetal bovine serum (FBS) (Sigma-Aldrich) and 10% DMSO (Sigma-Aldrich). Monocytes were isolated using the EasySep™ Human Monocytes Isolation Kit (Stemcell Technology) and cultured in RPMI-1640 (Lonza) containing 2 mM glutaMAX (Gibco), 20 µg/mL gentamicin (Gibco), and 2% normal human serum type AB (Invitrogen).

### Monocyte adhesion assay

HCAEC cell was cultured in DMEM medium supplemented with 10% FBS(Gibco), which had been recently authenticated and tested for mycoplasma contamination. Monocytes were cultured in RPMI-1640 medium supplemented with 10% sEV-free FBS(Gibco) and 1% penicillin-streptomycin. Cells were pelleted and resuspended to a concentration of 1 × 10^5^ cells/mL in a complete medium. Cells were labeled with 5 mmol/L Calcein-AM (Fisher Scientific, C3099) for 30 min. Monocytes were then layered over the HCAEC monolayers. After 1 h incubation, the nonadherent cells were washed off with PBS, and then the cells were fixed with 4% paraformaldehyde. Images were taken with an EVOS Fluorescence Microscope (Fisher Scientific), and the number of bound cells was manually counted with ImageJ.

### sEVs isolation

For sEVs isolation, monocytes were plated in 90 mm cell culture dishes using a medium supplemented with exosome-free fetal bovine serum. After incubation at 37 °C for 48 h, the cells were transferred to a starvation medium (medium without any fetal bovine serum) and cultured for 48 h. sEVs were isolated from starvation medium collected after 48 h of serum starvation using high-speed differential ultracentrifugation. Briefly, equivalent amounts of supernatant fractions were collected and pelleted by centrifugation at 500 g for 10 min to remove cells. The dead cells and cell debris were spun down from medium at 12,000 g for 20 min, and finally, sEVs were collected by centrifugation at 100,000 g for 70 min in a Beckman Type 45 Ti rotor using a Beckman L8-70 M Ultracentrifuge. The sEV pellets were resuspended in 20 mL of phosphate-buffered saline (PBS) and collected by ultracentrifugation at 100,000 g for 70 min in a Beckman Type 45 Ti rotor using a Beckman L8-70 M Ultracentrifuge, and the final pellet was resuspended in PBS.

### Density Gradient sEVs Isolation

In addition, sEVs were purified using an OptiPrep™ density gradient. Briefly, a discontinued iodixanol gradient was set by diluting a stock of OptiPrep™ (60% w/v) with 0.25 M sucrose/10 mM Tris, pH 7.5 to generate 40%, 20%, 10% and 5% w/v iodixanol solutions. The gradient was layered using 3 mL fractions each of 40%, 20%, 10%, and 5% w/v iodixanol solution. sEVs obtained after differential centrifugation was overlaid on the top of 5% w/v iodixanol solution and spun at 100,000 g at 4 °C for 18 h. Fractions of 1 mL were collected from the top of the tube and diluted with 1.5 mL of 1× PBS and further subjected to centrifugation at 100,000 g at 4 °C for 1 h. The pellet obtained was again washed with 1 mL 1× PBS and centrifuged at 100,000 g at 4 °C for 1 h to collect sEVs. The control OptiPrepTM gradient was run in parallel to determine the density of each fraction using 0.25 M sucrose/ 10 mM Tris, pH 7.5. The size distribution and concentration of sEVs were analyzed by nanoparticle-tracking analysis using a ZetaView particle tracker from Particle Metrix (Germany).

### Nanoparticle tracking analysis

Silica microsphere beads were used to calibrate a NanoSight LM10 (Malvern, UK). sEVs was diluted in PBS to produce 10^8^–10^9^ particles. Each sample was measured three times for 60 s, and the average value was used to determine the number of particles. The motion of each particle in the field of view was measured to produce the average displacement of each particle in a unit time, and the average displacement was calculated using NTA3.0 software (Nanosight).

### Transmission electron microscopy

A total of 10 μL of sEVs concentrate was placed on a Forvar-Carbon grid and dried for 20 min at room temperature. The enriched part was washed with PBS, fixed with 1% glutaraldehyde for 5 min, washed, and stained for 5 min with a uranyl oxalate saturated aqueous solution. The excess liquid was smeared on a piece of Whatman paper. The grid was then dried at room temperature for 10 min and imaged in a transmission electron microscope (FEI, USA).

### sEVs labeling

In the sEVs uptake experiment, the purified sEVs were labeled with a PKH67 green fluorescent cell-binding kit (Sigma-Aldrich). The sEVs separated by ultracentrifugation were then suspended in 1 ml PBS and marked with PKH67 dye according to the manufacturer’s instructions.

### RNA extraction and purification

Total RNA was extracted and purified using the exoRNeasy Serum/Plasma Midi Kit (Cat# 77044, QIAGEN, GmBH, Germany) following the manufacturer’s instructions and checked for a RIN number to inspect RNA integration by an Agilent Bioanalyzer 2100 (Agilent Technologies, Santa Clara, CA, US).

### RNA amplification and labeling

Total RNA was amplified and labeled by the Low Input Quick Amp Labeling Kit, One-Color (Cat.# 5190-2305, Agilent Technologies, Santa Clara, CA, US), following the manufacturer’s instructions. Labeled cRNA was purified by an RNeasy mini kit (Cat.# 74106, QIAGEN, GmBH, Germany).

### ChIP hybridization

Each slide was hybridized with 1.65 μg Cy3-labeled cRNA using a Gene Expression Hybridization Kit (Cat.# 5188-5242, Agilent Technologies, Santa Clara, CA, US) in a hybridization oven (Cat.# G2545A, Agilent Technologies, Santa Clara, CA, US) according to the manufacturer’s instructions. After 17 h of hybridization, the slides were washed in staining dishes (Cat.# 121, Thermo Shandon, Waltham, MA, US) with a Gene Expression Wash Buffer Kit (Cat.# 5188-5327, Agilent Technologies, Santa Clara, CA, US) following the manufacturer’s instructions.

### Data acquisition

Slides were scanned by an Agilent Microarray Scanner (Cat# G2565CA, Agilent Technologies, Santa Clara, CA, US) with the default settings: dye channel: green; scan Resolution=3 μm; PMT 100%; 20 bit. Data were extracted with Feature Extraction software 10.7 (Agilent Technologies, Santa Clara, CA, US). Raw data were normalized by the quantile algorithm, limma packages in R. The obtained circRNA microarray datasets were deposited with the NCBI Gene Expression Omnibus (GEO) repository under accession number GSE166126.

### ceRNA regulatory network construction

When we do ceRNA prediction analysis, miRNAs associated with differential or circRNA of interest were predicted by seed sequence matching analysis (circcinteractome (https://circinteractome.nia.nih.gov/) [29], circNet (http://circnet.mbc.nctu.edu.tw/), circbank (http://www.circbank.cn/help.html), deepBase (http://rna.sysu.edu.cn/deepBase/) [30]), circRNA-miRNA relationship pairs were found. Through miRTarBase (http://mirtarbase.cuhk.edu.cn/) [31], TargetScan (www.targetscan.org/), miRDB (mirdb.org/) [32], miRWalk (http://mirwalk.umm.uni-heidelberg.de/) [33], starbase2.0 and other online website predictions, miRNAs associated with differential or mRNAs of interest were predicted, and the mRNA-miRNA relationship pairs were found. The SCORE value was set to be greater than 0.8. The circRNA-miRNA relationship and the mRNA-miRNA relationship pairs were analyzed to obtain the common miRNA, and the correlation coefficient cor_xy analysis was performed on the circRNA/mirRNA and mRNA in the intersection to find a valid circRNA/mirRNA-mRNA relationship pair. The screening threshold (correlation coefficient) was set to greater than 0.8. We used cytoscape3.6.1 to visualize the relationship network. The greater the degree of connectivity, the larger the shape in the graph. Purple represents up-regulation, yellow represents down-regulation, and the circle represents mRNA, the diamond represents circRNA.

### Quantitative real-time PCR (qRT-PCR)

Total RNA was reverse-transcribed into cDNA with random primers using the Transcriptor First Strand cDNA Synthesis Kit (Roche, Penzberg, Germany) according to the manufacturer’s instructions. CircNPHP4 expression was quantified via qRT-PCR using FastStart Essential DNA Green Master Mix (Roche, Penzberg, Germany) on a Roche LightCycler 480 (Roche, Penzberg, Germany). Relative levels were determined using the 2^−ΔΔCt^ method, and the circRNA levels were normalized to the GAPDH levels. Divergent primers, rather than the more commonly used convergent primers, were designed to broadly target the circRNAs (Supplementary Table 2). Primer specificity was verified using BLAST, with a single peak in the melting curve indicating the generation of a specific product. Three experimental replicates were performed for each sample. Relative expression was determined using inter-experiment normalization to GAPDH. CircRNAs of the CAD sample with the lowest expression level were defined as 1.

### Plasmid constructs

CircNPHP4 was amplified from human genomic DNA and cloned into a pCD-ciR vector (Geenseed Biotech Co., Guangzhou, China) containing circRNA open reading frames. Site-directed mutagenesis was performed using a Fast Site-Directed Mutagenesis Kit (Takara Bio, Inc., Dalian, China) to target miRNA-binding sites on circNPHP4. All of the constructs were confirmed by sequencing.

### Western blotting

Proteins were extracted from a cell line and adipose tissues using RIPA lysis buffer. Protein concentrations were determined with a bicinchoninic acid protein assay kit (Sigma). The extracts were resolved via 12% SDS-PAGE and transferred to PVDF membranes. After blocking for 1 h, the membranes were incubated overnight at 4 °C with primary antibodies specific for: ICAM-1 (1:1000, Cell Signaling Technology, 67836 S), VCAM-1 (1:1000, Cell Signaling Technology, 39036 S), EGFR (1:1000, Cell Signaling Technology, 54359 S), AKT (1:1000, Cell Signaling Technology, 9272 S), p-AKT (1:1000, Cell Signaling Technology, 4060 S), PI3K (1:1000, Cell Signaling Technology, 4249 S), p-PI3K (1:1000, Abcam, ab278545), CD81(1:1000, Abcam, ab79559), CD63(1:1000, Abcam, ab134045), TSG101(1:1000, Abcam, ab125011). Next, the HRP-conjugated secondary antibody was added for 2 h at room temperature. The immunoreactive bands were visualized using ECL and normalized to GAPDH (the internal control).

### Fluorescent in situ hybridization

RNA fluorescent in situ hybridization (RNA-FISH) was performed following the instructions of the probe manufacturer’s instructions (RiboBio, Guangzhou, China; Supplementary Table 1). Monocytes were sequentially treated with 70%, 85%, and absolute ethanol and dried at 2 °C. Cells were then permeabilized with 0.1% Triton X-100 and incubated with a 20 μg/ml circNPHP4 probe overnight at 37 °C. The nuclei were stained with DAPI, and the intracellular localization of circNPHP4 was observed by using a TCS SP8 X laser confocal microscope (Leica).

### Luciferase reporter assay

A wild-type circNPHP4 sequence was cloned into a pmiR-RB-Report vector (RiboBio Co., Guangzhou, China) while simultaneously generating mutants using site-directed mutagenesis as described above. The mutations were confirmed by sequencing with vectors containing a mutation sequence used as a negative control. HCAECs were seeded in 96-well plates at a density of 4 × 10^3^ cells per well 24 h before transfection. The cells were then transfected with either the wild-type or mutated reporter vectors with lysates obtained 24 h post-transfection. The dual-luciferase assay was performed using the Dual-Glo Luciferase Reporter System (Promega, Madison, WI) according to the manufacturer’s protocols.

### Prediction of the miRNA-binding potential

Potential miRNA-binding sites on circNPHP4 were identified using the CircInteractome (https://circinteractome.nia.nih.gov/) predictive algorithm from the Shanghai Biotechnology Corporation (Shanghai, China). The identified miRNAs were then ranked based on their predicted binding scores.

### CircRNA immunoprecipitation (circRIP) assay

Biotin-labeled circNPHP4 probes were synthesized by GenePharma (Shanghai, China), and a circRIP assay was performed as previously described. Briefly, HCAECs were washed with ice-cold PBS, fixed using formaldehyde, lysed in co-IP buffer, and sonicated. After centrifugation, the supernatant was combined with streptavidin Dynabeads M-280 (Invitrogen, Waltham, MA, USA) and incubated at 30 °C for 12 h. Next, the probe-Dynabeads-circRNA mixture was washed and incubated with lysis buffer and proteinase K. Finally, the mixture was combined with TRIzol Reagent (Invitrogen, Carlsbad, CA, USA) for RNA extraction and detection.

### Biotin-coupled miRNA capture

The biotin-coupled miRNA pull-down assay was performed as previously described. Briefly, 3´-end biotinylated miR-1231 mimics (Ribio, Guangzhou, China) were transfected into monocytes for 48 h before harvest. The cell pellets were incubated with lysis buffer on ice. Then, streptavidin-coated magnetic beads (Life Technologies) were added to the cell lysates to pull down the biotin-coupled RNA complexes. The abundance of circNPHP4 in the bound fraction was evaluated by qRT-PCR analysis.

### RNA immunoprecipitation (RIP)

RIP experiments were performed using a Magna RIP RNA-Binding Protein Immunoprecipitation Kit (Millipore, Billerica, MA) according to the manufacturer’s instructions. An anti-AGO2 antibody (Cell Signaling Technology, Beverly, MA) was used for RIP. Coprecipitated circRNA was detected by qRT-PCR.

### Difference and enrichment analysis

We use the edgeR package of R software for difference analysis. The heat map and volcano map are drawn by the heatmap package and ggplot2 package of R software, respectively. Next, we used the org.Hs.eg.db package and cluster profile package for enrichment analysis.

### Mice

10 Male ApoE KO C57BL/6 J mice (15 weeks) were all purchased from GemPharmatech corporation and maintained under sterile conditions. Mice were randomly divided into 2 groups: ApoE KO + EGFRi group, ApoE KO mice were intragastrically administered Gefitinib (100 mg/kg) once daily and fed a high-fat diet (HFD; TD88137 Harlan Teklad), *n* = 5; ApoE KO group, ApoE KO mice were intragastrically administered an equivalent volume of DMSO once daily and fed an HFD, *n* = 5. The administration lasted 5 weeks. At the end of the experiment, the mice were euthanized for subsequent biochemical experiments. To visualize intracellular lipid deposits, Oil Red O staining was employed. Animal care and experimental procedures were approved by the Ethics Committee in Animal Experimentation of West China Hospital, Sichuan University, Chengdu, China (record #: 2019014 A).

### Statistical analyses

All statistical analyses were performed with SPSS v20.0 (SPSS, Inc., Chicago, IL). Data were presented as mean ± SD of at least three independent tests. Correlations between parameters were assessed using the Pearson correlation analysis. The student’s *t-*test was used to compare the two groups. Values of *P* < 0.05 were considered significant. Fisher’s exact test was used to identify significant correlations between circNPHP4 expression and clinicopathological features associated with the CAD patient samples. The calculation of the area under the curve (AUC) values were carried out according to the Receiver operating characteristic (ROC) curves. *P* < 0.05 was considered significant.

The least absolute shrinkage and selection operator (LASSO) method, which is suitable for reducing high-dimensional data [34, 35], was used to select the best predictive characteristics from among the risk factors for CAD. Features with nonzero coefficients in the LASSO regression model were selected [36]. Then, by combining the features selected in the LASSO regression model, a multivariate logistic regression analysis was used to build a predictive model. The 95% confidence interval (CI), odds ratio (OR), and *P*-value were considered. The level of statistical significance was a two-fold change. The statistical significance tests were all two-sided. Variables with a *P*-value less than 0.05 were included in the model, as were clinically important variables associated with the disease [37]. All potential predictors were used to develop a predictive model for the risk of CAD in the cohort (R packages “glmnet” and “rms”).

## Results

### sEVs from monocytes of CAD patients promoted heterogeneous adhesion

**Fig. 1:**
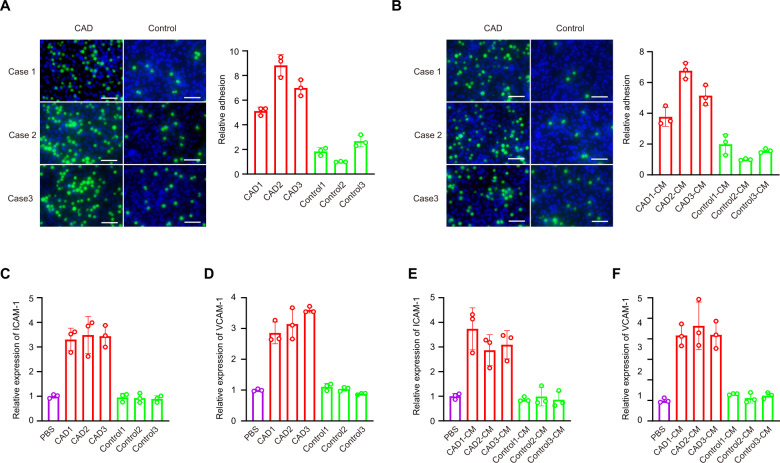
Heterogeneous adhesion ability of the CAD-derived monocytes was increased compared with the adhesion ability of the control monocytes. Heterogeneous adhesion ability of monocytes to HCAECs was analyzed using monocytes (**A**) or monocytes culture medium (CM) (**B**). ICAM-1 (**C**) and VCAM-1 (**D**) expression in HCAECs cocultured with monocytes were analyzed using qRT-PCR. ICAM-1 (**E**) and VCAM-1 (**F**) expression in HCAECs cocultured with monocytes culture medium were analyzed using qRT-PCR. Scale bar = 50 µm. Data are presented as means ± SD; a significant difference was identified with Student’s *t* test. *P < 0.05; **P < 0.01. ns (not significant).

Using the EasySep Human CD14 and CD16 Selection Kit, we isolated CD14 + CD16 + monocytes from PBMCs (Supplementary Fig. 1). Our results showed that the heterogeneous adhesion ability of the CAD-derived monocytes to HCAECs was increased compared with the adhesion ability of the control monocytes to HCAECs (Fig. 1A). Moreover, heterogeneous adhesion ability increased between control monocytes and HCAECs cocultured with culture medium (CM) from coronary atherosclerotic monocytes (Fig. 1B). To investigate the mechanism of altered monocyte adhesion, we measured the levels of two adhesion proteins, ICAM-1 and VCAM-1, in HCAECs induced with TNF-α using qRT-PCR and western blotting. Our results showed that ICAM-1 and VCAM-1 expression in HCAECs cocultured with monocytes from CAD patients was significantly upregulated compared with expression in HCAECs cocultured with control monocytes (Fig. 1C, D and Supplementary Fig. 2A). Parallel ICAM-1 and VCAM-1 expression results were obtained using CM from CAD-related monocytes (Fig. 1E, F and Supplementary Fig. 2B).

**Fig. 2:**
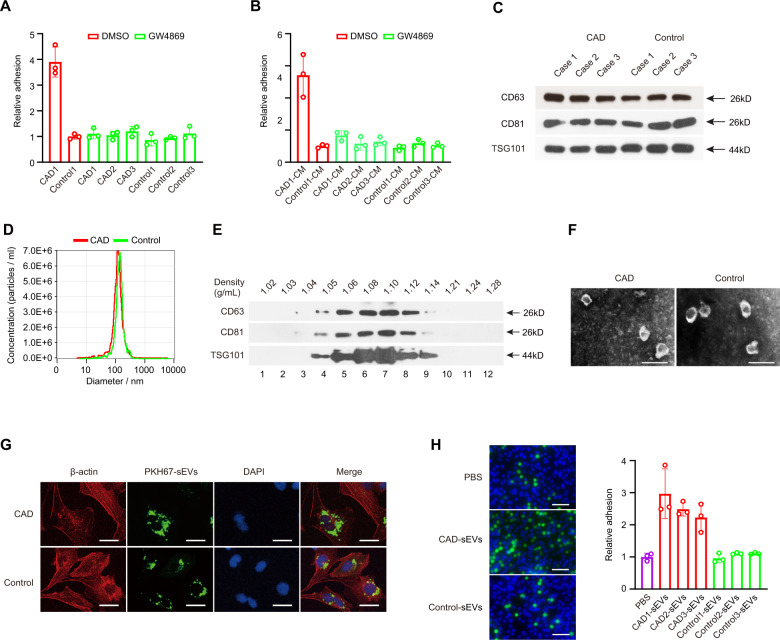
sEVs from monocytes of CAD patients promoted heterogeneous adhesion. Heterogeneous adhesion ability of monocytes to HCAECs was analyzed using monocytes (**A**) or monocytes culture medium (CM) (**B**) following GW4869 exposure. sEVs from monocytes were extracted to be verified using western blot assay (**C**) and the NTA method (**D**). CD63, CD81, and TSG101 expression were detected in fractions collected from OptiPrep^TM^ density gradient centrifugation using western blot assay (**E**). sEVs obtained from fraction 7 (density 1.10 g/mL) was verified using transmission electron microscopy (**F**). Scale bar = 200 nm. **G** HCAECs were incubated directly with sEVs from CAD patients or control-related monocytes. Scale bar = 20 µm. **H** Heterogeneous adhesion ability of monocytes to HCAECs was analyzed using CAD-sEVs or Control-sEVs. Scale bar = 20 µm. Data are presented as means ± SD; a significant difference was identified with Student’s *t* test. *P < 0.05; **P < 0.01; ns (not significant).

These data suggested that monocytes endow HCAECs with an enhanced heterogeneous adhesion capacity through their secreted factors. Monocyte-derived sEVs could be transferred to target cells to alter the function of these cells [38]. To further verify the sEV function in the heterogeneous adhesion capacity between monocytes and HCAECs, the sEVs inhibitor neutral sphingomyelinase (nSMase2), GW4869 [39], was used to treat monocytes for 24 h before HCAECs were cocultured with monocytes. There was no difference in heterogeneous adhesion capacity between CAD-related monocytes and control monocytes to HCAECs (Fig. 2A), and parallel results of the heterogeneous adhesion ability were obtained when cocultured in CM from monocytes exposed to GW4869 (Fig. 2B).

We next extracted sEVs from monocytes. Using a western blot assay, we showed that protein markers of sEVs CD63, CD81, and TSG101 were highly expressed in sEVs from monocytes (Fig. 2C). Next, we detected the sizes of sEVs by the NTA method and found that the sizes of sEVs from monocytes were between 40 and 150 nm (Fig. 2D), which was consistent with common sizes of known sEVs [40]. Calnexin which is considered as a negative control for sEV preparations, could not be seen in Western blot assay (Supplementary Fig. 3). In addition, sEVs were purified using an OptiPrep™ density gradient. Fractions of increasing density were collected, and Western blot analysis was performed to identify sEVs enriched samples. As shown in Fig. 2E, monocytes-derived sEVs were enriched in fractions 6–8 corresponding to density 1.08–1.12 g/mL. This density is consistent with previously reported studies conducted on different cell types [41, 42]. Next, transmission electron microscopy of sEVs obtained from fraction 7 corresponding to density 1.10 g/mL revealed vesicles that were consistent with the size and morphology of sEVs (Fig. 2F). Thus, we successfully extracted sEVs secreted by monocytes isolated from CAD patients and control.

We further examined whether sEVs from CAD-related monocytes could promote the heterogeneous adhesion ability between control monocytes and HCAECs. In a coculture adhesion assay, HCAECs were incubated directly with sEVs from CAD-related monocytes (CAD-sEVs) and sEVs from monocytes given by healthy donors (Control-sEVs) for 48 h. We used a confocal microscope to analyze the recipient cells and found a strong green fluorescence in the cytoplasm of recipient cells after incubating the cells with PKH67-labeled sEVs isolated from CAD-related monocytes (Fig. 2G). Moreover, compared with Control-sEVs, CAD-sEVs could significantly promote the heterogeneous adhesion between monocytes and HCAECs (Fig. 2H), therefore confirming that sEVs might play an important role in heterogeneous adhesion between HCAECs and monocytes.

### CircRNA expression profile in sEVs from CAD patient monocytes

**Fig. 3:**
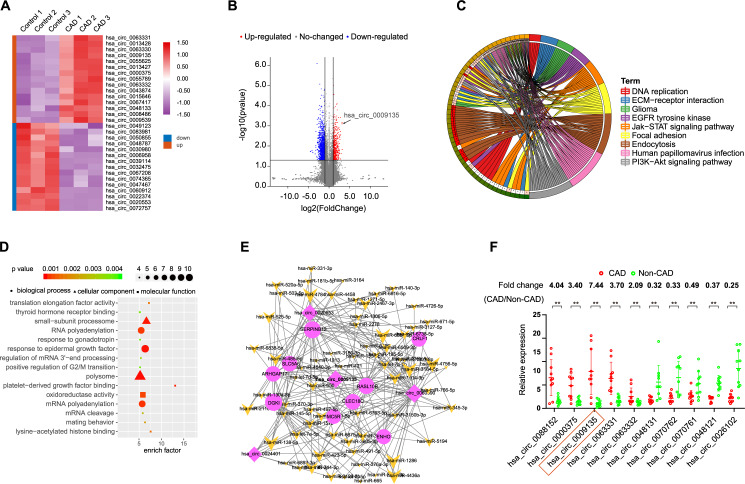
Identification of differentially expressed circRNAs in sEVs from CAD patient monocytes. **A** Clustered heatmap of the differentially expressed circRNAs in sEVs from monocytes. Upregulated circRNAs are shown in red and downregulated circRNAs are shown in green. **B** Volcano plots comparing circRNA expression between CAD patient and control. The red dots represent the significantly differentially expressed circRNAs (fold-change ≥1.5 and *P* < 0.01). **C** Top 15 classes of KEGG pathway enrichment terms. **D** Top 15 classes of disease enrichment terms (**E**) CircRNA-miRNA-mRNA network and pathway analysis. **F** The differential expression of 10 circRNAs in sEVs was validated in 10 monocytes from CAD patients and 10 monocytes from control using qRT-PCR. Data are presented as means ± SD; significant difference was identified with Student’s *t* test. *P < 0.05; **P < 0.01; ns (not significant).

To investigate the function of monocyte-derived circRNAs in heterogeneous adhesion in CAD, sEVs from the 3 patients and control sEVs from 3 healthy persons were analyzed for circRNA expression profiles using circRNA microarrays. The circRNA profile dataset was visualized using a heatmap (Fig. 3A). After normalization, log2 ratio distributions were examined among the six samples and were very similar. Significantly differentially expressed circRNAs between the two groups were identified using volcano plot filtering (Fig. 3B). In the CAD samples, 310 significantly differentially expressed circRNAs were identified relative to the control samples (*P* < 0.01) (Supplementary Table 3), with 112 upregulated and 198 downregulated circRNAs. Both functional enrichment analysis and KEGG signaling pathway analysis also suggested that the abnormal expression of circRNAs was associated with the EGFR pathway (Fig. 3C, D). We also constructed a network containing circRNA-miRNA-mRNA pathways, which might be responsible for the regulation of the biological mechanisms underlying CAD (Fig. 3E).

### Characterization of circNPHP4 in monocyte

**Fig. 4:**
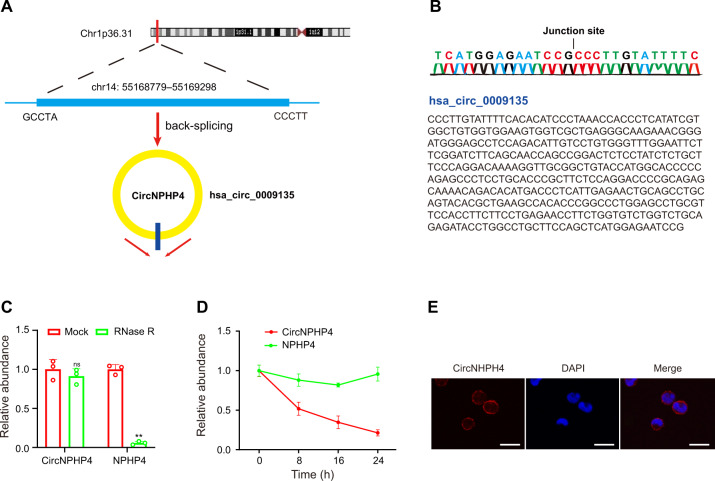
Characterization of circNPHP4 in monocytes. **A** The genomic location of the hNPHP4 gene and of circNPHP4. **B** Sanger sequencing showing the “head-to-tail” splicing of circNPHP4 in the monocytes. **C** qRT-PCR quantification of circNPHP4 and hNPHP4 mRNA expression in monocytes after treatment with RNase R. **D** qRT-PCR quantification of circNPHP4 and hNPHP4 mRNA expression in monocytes after treatment with Actinomycin D. Data are presented as means ± SD; significant difference was identified with Student’s *t* test. *P < 0.05; **P < 0.01; ns (not significant). **E** RNA FISH for circNPHP4. Nuclei were stained with DAPI. Scale bar = 20 µm.

After examining the obtained microarray data, the top 5 upregulated differentially expressed circRNAs as well as the top 5 downregulated circRNAs, as defined by having the most significant *P*-value, were examined further (Supplementary Table 3). These circRNAs were further examined using qRT-PCR; CAD sEVs samples from 10 patients and control sEVs samples from 10 healthy people were examined (Fig. 3F). The most significantly expressed circRNA was hsa_circ_0009135, and based on the human reference genome (GRCh37/hg19), we hypothesized that hsa_circ_0009135 (chr14: 55168779–55169298) is derived from NPHP4, which is located on chromosome 14q22 (Fig. 4A). Thus, hsa_circ_0009135 was named “circNPHP4.” The sequence of circNPHP4 was confirmed with Sanger sequencing (Fig. 4B), and it was found to be RNase R resistant, thus suggesting a circular configuration (Fig. 4C). To measure the half-life of circNPHP4 and NPHP4, the levels were examined following actinomycin D transcriptional inhibition, and circNPHP4 was found to be more stable than NPHP4 (Fig. 4D). Furthermore, examination with RNA-FISH showed that circNPHP4 is predominantly located in the cytoplasm of monocytes (Fig. 4E). Moreover, our results showed that circNPHP4 levels in sEVs were almost equal to that in the whole CM (Supplementary Fig. 4), indicating that sEV was the main carrier for extracellular circNPHP4.

### CircNPHP4 from CAD-related monocyte-derived sEVs promoted heterogeneous adhesion

**Fig. 5:**
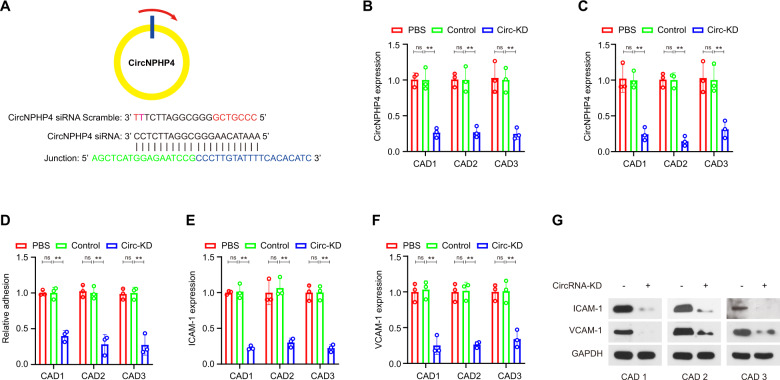
CircNPHP4 from monocyte-derived sEVs promoted heterogeneous adhesion. **A** Schematic representation of the siRNA sites specific to the back-splice junction of circNPHP4. Expression of circNPHP4 following siRNA treatment using qRT-PCR in monocytes (**B**) and in sEVs from monocytes (**C**). Heterogeneous adhesion was analyzed after transfection with circNPHP4 specific siRNA versus scramble controls (**D**). ICAM-1 (**E**) and VCAM-1 (**F**) were detected in HCAECs exposed to sEVs from circNPHP4-knockdown monocytes, as quantified by qRT-PCR and western blot analyses (**G**). Data are presented as means ± SD; a significant difference was identified with Student’s *t* test. *P < 0.05; **P < 0.01; ns (not significant).

CircNPHP4 knockdown was performed using siRNAs targeting the back-splice sequence in monocytes (Fig. 5A). Successful knockdown was confirmed using qRT-PCR in both sEVs and monocytes (Fig. 5B, C). Furthermore, in a coculture adhesion assay, HCAECs incubated directly with sEVs derived from circNPHP4-downregulated monocytes from CAD patients showed significantly decreased heterogeneous adhesion ability (Fig. 5D). Additionally, heterogeneous adhesion biomarkers, ICAM-1 and VCAM-1, were decreased in HCAECs exposed to sEVs from circNPHP4-knockdown monocytes, as quantified by qRT-PCR and western blot analyses (Fig. 5E, F, G).

### CircNPHP4 may function as a sponge for miR-1231 in HCAECs

**Fig. 6:**
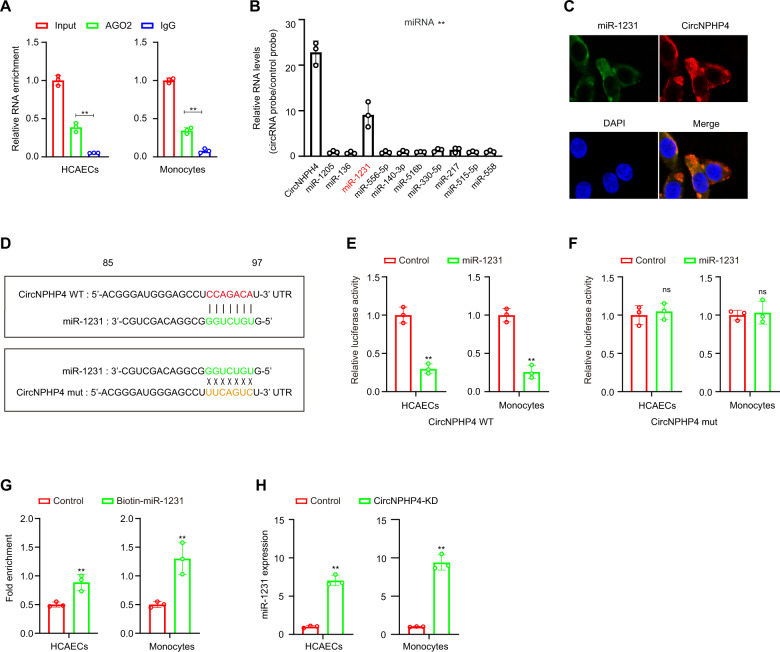
CircNPHP4 acted as a miRNAs sponge for miR-1231. **A** RIP was performed using an antibody against AGO2 on extracts from monocytes and HCAECs. **B** CircNPHP4 was performed using a circNPHP4-specific probe and control probe in HCAECs. The enrichment of circNPHP4 and microRNAs were detected by qRT-PCR and normalized to the control probe. **C** Co-localization between circNPHP4 and miR-1231 was observed by RNA in situ hybridization in HCAECs. Nuclei were stained with DAPI. Scale bar = 20 µm. **D** Schematic showing the predicted miR-1231 sites in circNPHP4. A Luciferase assay where monocytes were co-transfected with a scrambled control, miR-1231 mimic, and a luciferase reporter plasmid containing wild-type circNPHP4 (circNPHP4-WT) (**E**) or mutant circNPHP4 (circNPHP4-mut) (**F**). **G** qRT-PCR showed the level of circNPHP4 in the streptavidin-captured fractions from the monocytes and HCAECs lysates after transfection with biotinylated miR-1231 or control RNA. **H** Expression of miR-1231 was analyzed using qRT-PCR following circNPHP4 knockdown. Data are presented as means ± SD; a significant difference was identified with Student’s *t* test. *P < 0.05; **P < 0.01; ns (not significant).

Previous studies have shown that circRNAs act as miRNA sponges [43]; thus, the ability of circNPHP4 to bind to miRNAs was explored. To identify miRNAs that might potentially bind to circNPHP4, CircInteractome was employed and identified 10 potential miRNAs (Supplementary Table 4). To further explore circNPHP4 association, a circRIP assay with antibodies against argonaute 2 (AGO2) was chosen due to AGO2 having a role in miRNA-induced RNA silencing in adipose tissue [44]. The results showed that the anti-AGO2 antibody significantly enriched circNPHP4 (Fig. 6A), thus suggesting that circNPHP4 may act as a binding platform for AGO2 and miRNAs. Additionally, circNPHP4-associated miRNAs were purified using circRIP with specific probes targeting circNPHP4. The results showed that circNPHP4 and miR-1231 were both enriched in the examined monocytes (Fig. 6B). This association was further confirmed using RNA-FISH and showed that circNPHP4 and miR-1231 are colocalized in HCAECs (Fig. 6C).

To examine the predicted circNPHP4-binding sites (Fig. 6D), a dual-luciferase assay was performed. The results showed that high binding affinity occurred between circNPHP4 and miR-1231. Furthermore, compared to the control group, the miR-1231 group had an obviously reduced luciferase reporter activity (Fig. 6E). When mutating the miR-1231 target sites in the luciferase reporter, no significant change in luciferase activity was noted following transfection with miR-1231 and the luciferase reporter (Fig. 6F). Furthermore, by a pull-down assay using a biotin-coupled miR-1231 mimic, we observed that circNPHP4 in the mimic group was obviously enriched compared with that in the control group, while the cicrANRIL group (negative control) had no enrichment (Fig. 6G, H). Thus, our results suggest that sEV-derived circNPHP4 knockdown in CAD-derived monocytes suppresses heterogeneous adhesion possibly by reducing the functionality of miR-1231.

### CircNPHP4 may promote the EGFR/PI3K/AKT pathway through miR-1231 inhibition in HCAECs

**Fig. 7:**
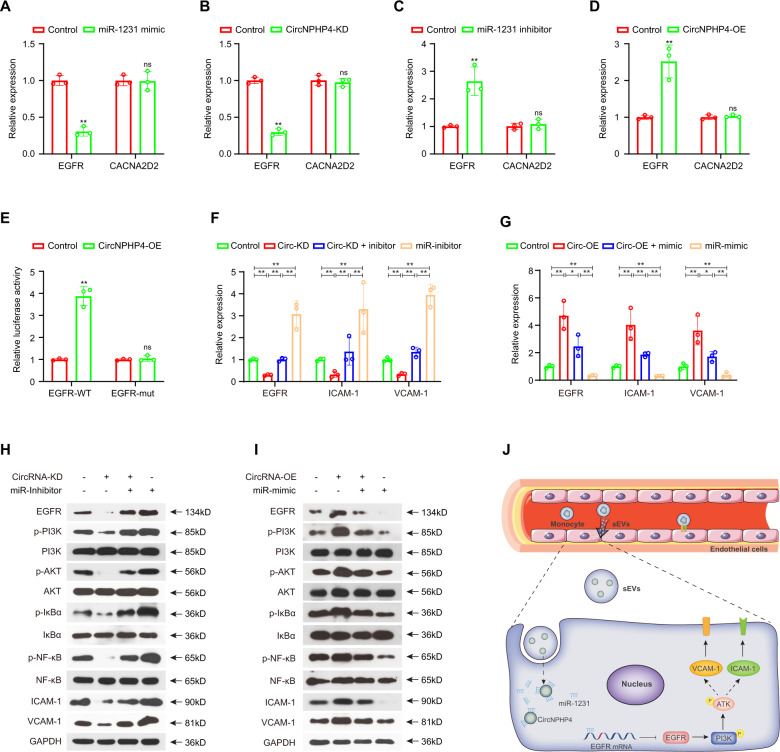
CircNPHP4 promoted the EGFR/PI3K/AKT pathway through miR-1231. CircNPHP4 promoted the EGFR/PI3K/AKT pathway through miR-1231 inhibition in HCAECs qRT-PCR quantification of EGFR and CACNA2D2 expression after miR-1231 overexpression (**A**) or circNPHP4 knockdown (**B**). EGFR and CACNA2D2 expression after transfection with miR-1231 inhibitor (**C**) or circNPHP4 expression vector (**D**) was quantified with qRT-PCR. Luciferase assay where HCAEC were co-transfected with a scrambled control, circNPHP4 expression plasmid, and a luciferase reporter plasmid containing either wild-type EGFR (EGFR-WT) or an EGFR construct with mutated miR-1231 binding sites (EGFR-mut) (**E**). **F–I** Reversion assays using vectors overexpressing or knocking down circNPHP4, as well as miR-1231 mimics or inhibitors. Data are presented as means ± SD; a significant difference was identified with Student’s *t* test. *P < 0.05; **P < 0.01; ns (not significant). **J** A proposed model illustrating the role of monocyte-derived sEVs circNPHP4 in regulating heterogeneous adhesion in vein endothelial cells.

Previous reports showed that miR-1231 could target EGFR and CACNA2D2 directly in glioma and embryonic kidney cells [45, 46]. Thus, we hypothesized that exosomal circNPHP4 induces heterogeneous adhesion of HCAECs by protecting the EGFR signaling pathway or CACNA2D2 from downregulation by miR-1231. To test this hypothesis, we overexpressed miR-1231 mimics and measured the expression of their respective targets via qRT-PCR. Following mimic transfection into HCAECs, EGFR, but not CACNA2D2, was found to exhibit a significant decrease in gene expression (Fig. 7A). Furthermore, *circNPHP4* knockdown significantly reduced EGFR expression (Fig. 7B), while *circNPHP4* overexpression or miR-1231 inhibition increased EGFR expression (Fig. 7C, D).

Next, the 3´UTR of EGFR was cloned into a luciferase vector, and the effect of miR-1231 in the presence or absence of *circ*NPHP4 was examined. In HCAECs overexpressing *circ*PHP4, those containing the wild-type EGFR 3´UTR had a higher luciferase reporter activity than the controls but did not have a higher activity than those containing a mutated EGFR 3´UTR (Fig. 7E). However, in cells with circNPHP4 knockdown and miR-1231 inhibition, the EGFR expression levels and downstream *PI3K/AKT* pathway activity were significantly rescued (Fig. 7F, H). It has been shown that upon stimulation with cytokines, IκB-α undergoes phosphorylation and subsequent degradation, thereby allowing translocation of NF-κB p65 to the nucleus, where it can initiate transcription of many target genes, including VCAM-1 and ICAM-1 [47, 48]. Moreover, the *PI3K/AKT* pathway could activate the NF-κB pathway [49, 50], which changed with the variation in the circNPHP4 and miR-1231 levels. Furthermore, miR-1231 upregulation significantly reduced the EGFR levels upon circNPHP4 overexpression (Fig. 7G, I). As proteins downstream of the PI3K/AKT/ NF-κB pathway [51, 52], ICAM-1 and VCAM-1 expression changed with the variation in the circNPHP4 and miR-1231 levels upon knockdown. Moreover, parallel results of EGFR/PI3K/AKT pathway activity by circNPHP4 were obtained in Human Umbilical Vein Endothelial Cells (HUVECs) (Supplementary Fig. 5). These data suggest that circNPHP4 induces heterogeneous monocyte adhesion by interacting with miR-1231 within the circNPHP4/miR-1231/EGFR axis (Fig. 7J).

### CircNPHP4 upregulation in serum predicts aggressive clinicopathological characteristics

**Fig. 8:**
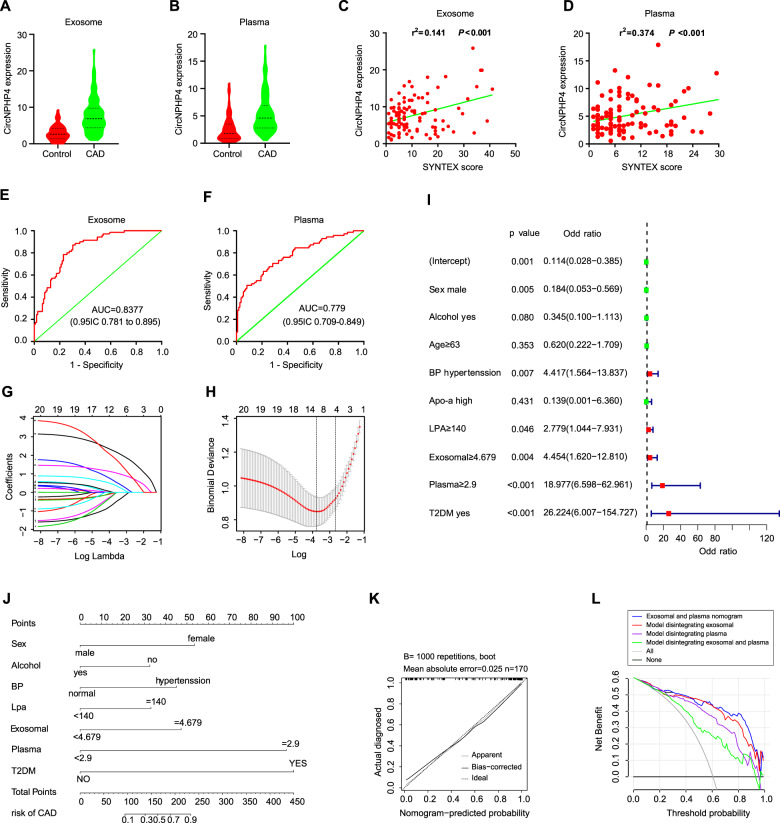
CircNPHP4 upregulation in serum predicts aggressive clinicopathological characteristics. **A** CircNPHP4 expression in serum from 109 CAD patients and 70 control. **B** CircNPHP4 expression in serum from 109 CAD patients and 70 control. **C** Pearson correlation between circNPHP4 expression and SYNTEX score in serum of CAD patients. **D** Pearson correlation between circNPHP4 expression and SYNTEX score in serum of CAD patients. **E** ROC curve for serum circNPHP4 that indicates a diagnostic value in CAD patients. **F** ROC curve for serum circNPHP4 that indicates a diagnostic value in CAD patients. **G** Lambda in the CAD and control cohort. **H** LASSO coefficient profiles of the 20 features. A coefficient profile plot was produced against the log (lambda) sequence. A vertical line was drawn at the value selected using fivefold cross-validation, where optimal lambda resulted in 9 features with nonzero coefficients. **I** Logistic regression analysis in the CAD and control cohort. **J** Nomogram using the circNPHP4 upregulation to estimate the diagnosis rate of CAD. **K** Calibration curves of the diagnosis prediction nomogram in the primary cohorts. **L** Decision curve analysis for the CAD nomogram in the cohort. The y-axis measures the net benefit. The black line represents the CAD risk nomogram. The thin solid line represents the assumption that all patients have CAD. The thick solid line represents the assumption that all patients are in Non-CAD. The decision curve shows that if the threshold probability of a patient and a doctor is >2% and <96%, respectively, using this CAD nomogram in the current study to predict CAD risk adds more benefit than the scheme.

As circNPHP4 expression was significantly increased in sEVs from CAD-related monocytes and sEVs could be secreted by monocytes into the serum, we next analyzed whether circNPHP4 could be detected in the serum and was correlated with the clinicopathological status of coronary artery disease (CAD) patients. We used qRT-PCR to quantify circNPHP4 expression in a cohort of 109 CAD patients and 70 control (Supplementary Table 5). CircNPHP4 expression in the serum from CAD patients was significantly upregulated compared to that in the serum from control patients (Fig. 8A). Parallel results of the expression of sEV-derived circNPHP4 were obtained using serum from CAD patients and the control group (Fig. 8B). We also analyzed the association between circNPHP4 expression and the clinicopathological status of CAD patients (Supplementary Table 6). CircNPHP4 expression using serum or serum sEVs was positively correlated with the SYNTX scale (*P* < 0.01) in CAD patients (Fig. 8C, D). Furthermore, ROC analysis was performed with a circNPHP4 cutoff ≥ 5.041 and showed a high diagnostic performance using serum sEVs, as reflected by the Youden index (sensitivity, 87.1%; specificity, 69.7%; Fig. 8E). Parallel results of ROC analysis were obtained using serum samples (Fig. 8F).

To overcome the limitations of probabilistic prediction models, we constructed a feature selection and developed an individualized predictive model that can be used to facilitate medical decisions based on the decision maker’s preferences. First, there were 9 potential predictors in the cohort (Fig. 8G, H) that had nonzero coefficients in the LASSO regression model: age, sex, blood pressure, Apo-a, LPA, alcohol consumption history, exosomal circNPHP4 expression, serum circNPHP4 expression and type 2 diabetes mellitus (T2DM). The results of the logistic regression analysis are given in Fig. 8I. Next, the model that incorporated the above independent predictors was developed and is presented as a nomogram (Fig. 8J). Our results also showed that the calibration curve of the nomogram for the prediction of the risk of CAD demonstrated good agreement (Fig. 8K). The C-index for the predictive nomogram was 0.927 (95% confidence interval: 0.917-0.937) for the primary cohort, and a C-index of 0.921 was confirmed with bootstrapping validation, which suggests that the nomogram has a good discriminatory ability. The performance parameters of the nomogram for the risk of CAD had a good predictive capability.

We also performed decision curve analysis for the CAD nomogram, as shown in Fig. 8L. The decision curve showed that if the threshold probabilities of a patient and a doctor were >2% and <96%, respectively, using this CAD nomogram to predict the risk of CAD added more benefit than the scheme. In addition, exosomal and serum circNPHP4 expression levels are indispensable factors in the predictive model. When one or both of them are missing, the benefit obtained by using the model significantly decreased. Moreover, compared with the exosomal circNPHP4 expression level, the contribution of the serum circNPHP4 expression level to the model was greater.

## Discussion

CircRNAs are emerging as an important new class of regulators that impinge on diverse biological processes and the pathogenesis of CAD. Some circRNAs have been found to be associated with heterogeneous adhesion between monocytes and endothelial cells, and the altered expression of circRNAs was correlated with the occurrence and development of CAD [53]. In the present study, we found a novel regulator of heterogeneous adhesion between monocytes and HCAECs, circNPHP4, which is upregulated in sEVs from the monocytes of CAD patients and predicts aggressive clinicopathological characteristics. Furthermore, exosomal circNPHP4 promotes heterogeneous adhesion and acts as a ceRNA by sponging miR-1231 to modulate EGFR signaling pathway activation.

Due to its important role in CAD, the circRNA profile of CAD patients has been assessed by microarray in PBMCs [54]. The study showed that circRNA hsa_circ_0001879 and hsa_circ_0004104 are biomarkers for coronary artery disease. In the present study, we found that these circRNAs were more highly expressed in CAD-derived monocytes than in control monocytes (Supplementary Fig. 6) but were not highly expressed in sEVs from CAD patients and control monocytes, which indicated that these circRNAs could not be secreted into sEVs from monocytes. In my research, circNPHP4 was the most highly expressed circRNA in sEVs from CAD-derived monocytes. Recent research revealed that serum exosomal hsa_circ_0124644 [42], hsa_circ_0001879 and hsa_circ_0004104 [54] might be novel circRNA biomarkers to diagnose CAD. Compared with these circRNAs, circNPHP4 had the better diagnostic efficiency with a higher AUC value in our study. Moreover, serum exosomal circNPHP4 expression level is indispensable factors in the CAD nomogram predictive model.

It has been reported that circNPHP4 can be detected in human epidermal keratinocyte cells [55] and foreskin fibroblasts [56]. In the present study, circNPHP4 was significantly upregulated in sEVs from CAD-related monocytes compared with control monocytes. We found that circNPHP4 was derived from an exon of NPHP4, which is a partner of the nephrocystin multimolecular signaling complex and might regulate cell-cell and cell-matrix adhesion. Additionally, circNPHP4 expression was positively correlated with NPHP4 expression in monocytes from CAD patients and control (Supplementary Fig. 7).

Furthermore, we constructed a circRNA-miRNA interaction network to identify the most informative circRNA candidates. It was hypothesized that functionally dysregulated circRNAs could effectively capture target miRNAs and modulate their activity [57]. Following miRNA targeting and circRIP analyses, our findings suggested that circNPHP4 might act as a miRNA sponge by interacting with miR-1231. In monocytes, RNA-FISH analysis showed that circNPHP4 and miR-1231 were colocalized. These findings supported the notion that circRNAs can affect gene expression by acting as a “miRNA sponge”. Although circNPHP4 was already bound to miR-1231 within monocytes, the expression of circNPHP4 in sEVs was higher than that of miR-1231 (Supplementary Fig. 8). These results suggested circNPHP4 could bind miR-1231 in HCAECs. Additionally, while examining the structure of circNPHP4, we found an internal ribosome entry site (IRES), which can possibly induce 5’-cap-independent translation (Supplementary Fig. 9), suggesting its translational potential. While our findings indicated that circNPHP4 acted as a miRNA sponge, other potential functions still require further elucidation.

This study has some limitations that must be noted. Because the screening was performed in a small set of samples, differential expression circRNAs should be validated in larger cohorts. Due to the lack of follow-up information for CAD patients, the prognostic value of circNPHP4 in major cardiovascular events should be evaluated in subsequent studies. However, circNPHP4 is not conserved in mice, so we analyzed the mechanisms underlying the regulation of differentiation in vivo using EGFR inhibitor Gefitinib. We found significantly smaller aortic lesions in these mice than in HFD-treated mice. Oil-red-O-stained aortic sinus in EGFR inhibitor (Gefitinib)-treated ApoE KO mice did not show any significant increase in lipid content relative to control ApoE KO mice (Supplementary Fig. 10).

The mechanisms underlying the regulation of differentiation in vivo still require further study. Compared with RNA-seq, circRNA microarray does not allow for de novo circRNA identification for the principle of designing probes based on sequences of existing circRNAs.

Overall, this study identified more than 300 circRNAs in sEVs derived from monocytes of CAD patients using circRNA microarray analysis. Of the identified circRNAs, circNPHP4 expression was significantly upregulated, and this increased expression was correlated with aggressive clinicopathological characteristics in CAD patients. Furthermore, the circNPHP4-miR-1231-EGFR axis was shown to affect heterogeneous adhesion between sEVs and HCAECs in CAD patients. These results provide a foundation for further functional, diagnostic, and therapeutic studies related to circRNAs in CAD patients.

## Supplementary information

supplementary figures, tables, and code

Supplementary Table 1

## Data Availability

The obtained circRNA microarray datasets were deposited with the NCBI Gene Expression Omnibus (GEO) repository under accession number GSE166126. Other data and code used in this study are provided in the supplementary file. All data is accessible.
