# Supplementary material for: CircNPHP4 in monocyte-derived small extracellular vesicles controls heterogeneous adhesion in coronary heart atherosclerotic disease
Source: Cell Death Dis. 2021 Oct 14;12(10):948. doi: 10.1038/s41419-021-04253-y (PMC8516978; doi:10.1038/s41419-021-04253-y)
Supplement: Supplementary file 1 — supplementary figures, tables, and code [file 41419_2021_4253_MOESM1_ESM.docx]

**Supplementary Figures**:


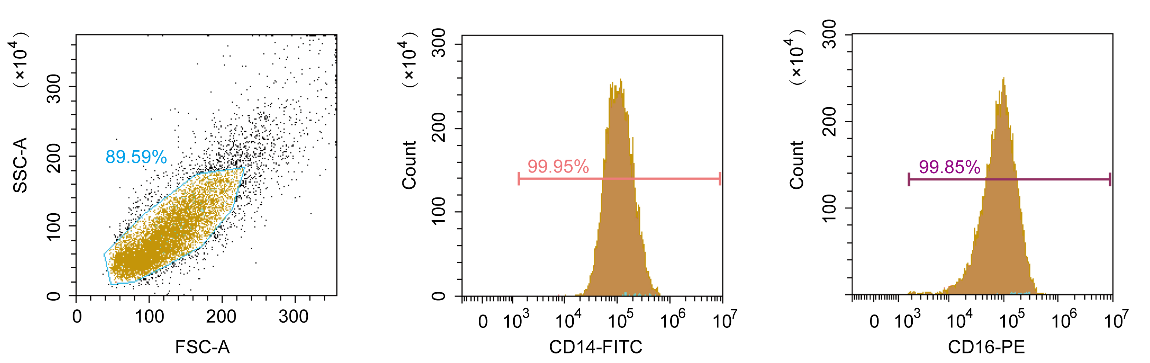


Supplementary Fig. S1. Monocytes purification from PBMCs. CD14 and CD16 expression in monocytes were analyzed by flow cytometry.


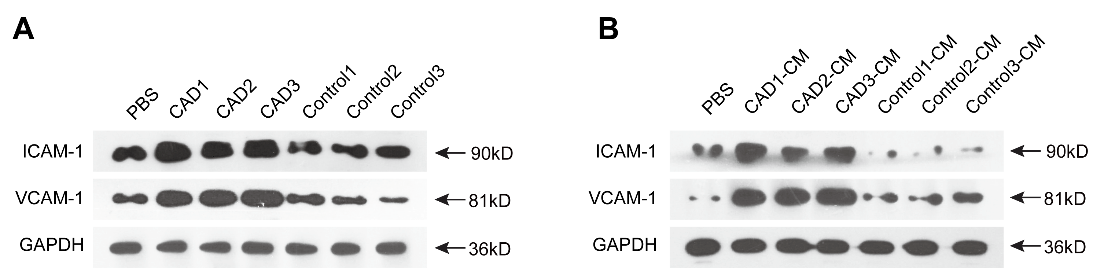


Supplementary Fig. S2. ICAM-1 and VCAM-1 expression in HCAECs cocultured with monocytes (A) or culture medium (B) from CAD patients was detected using western bolt assay.


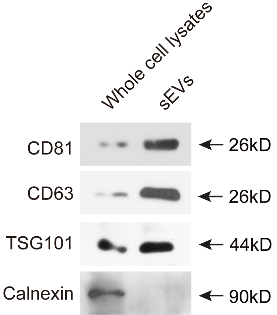


Supplementary Fig. S3. Calnexin expression was detected in sEVs or whole cell lysates from monocytes using western blot analysis.


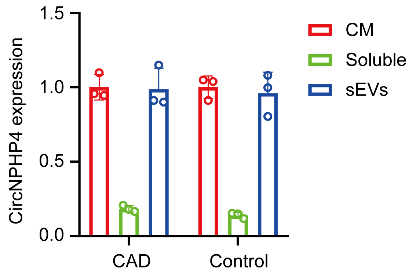


Supplementary Fig. S4. qRT-PCR analysis of circNPHP4 in sEVs, soluble fraction of CM (soluble) and whole CM derived from monocytes. Data are presented as means ± SD; significant difference was identified with Student's t test. *P < 0.05; **P < 0.01; ns (not significant).


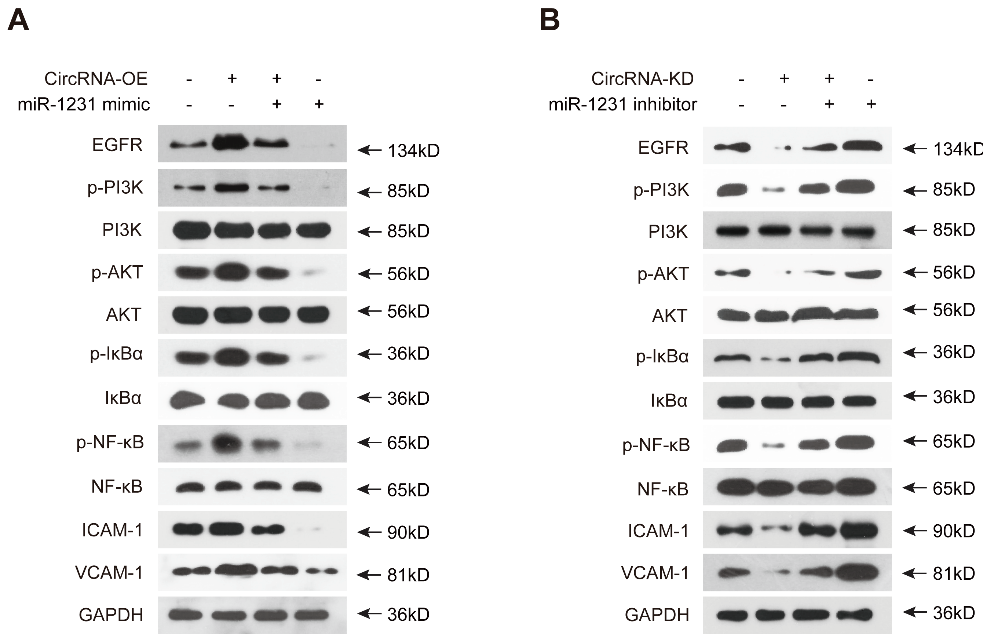


Supplementary Fig. S5. Reversion assays using vectors overexpressing (A) or knocking down (B) circNPHP4, as well as miR-1231 mimics or inhibitors in HUVECs through western blot assay.


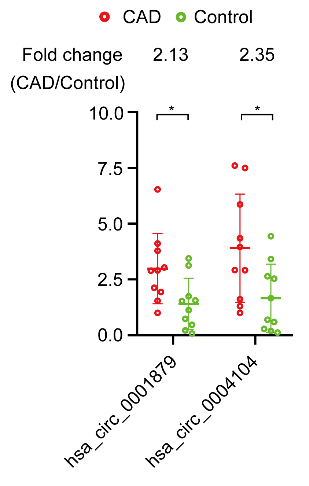


Supplementary Fig. S6. hsa_circ_0001879 and hsa_circ_0004104 expression was detected in monocytes from CAD patients and control using qRT-PCR analysis. Data are presented as means ± SD; significant difference was identified with Student's t test. *P < 0.05; **P < 0.01; ns (not significant).


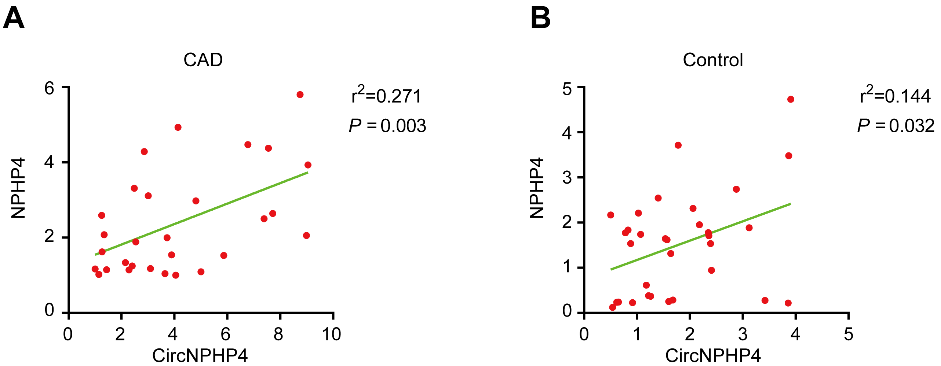


Supplementary Fig. S7. Correlation analysis between circNPHP4 and NPHP4 expression was conducted in monocytes from CAD patients and control using qRT-PCR. Data are presented as means ± SD; significant difference was identified with Student's t test. *P < 0.05; **P < 0.01; ns (not significant).


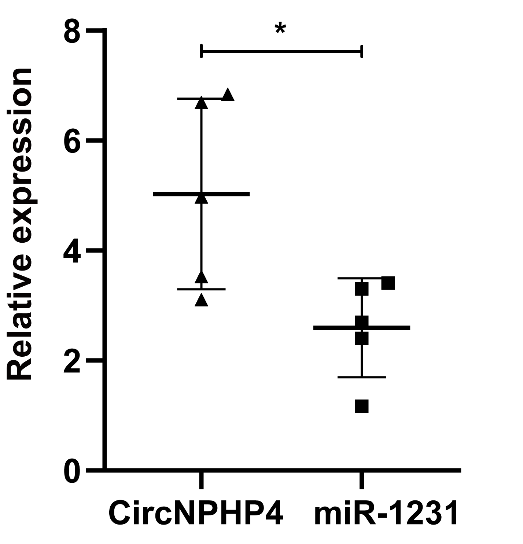


Supplementary Fig. S8. The expression of circNPHP4 and miR-1231 in sEVs was detected by qRT-PCR. Data are presented as means ± SD; significant difference was identified with Student's t test. *P < 0.05; **P < 0.01; ns (not significant).


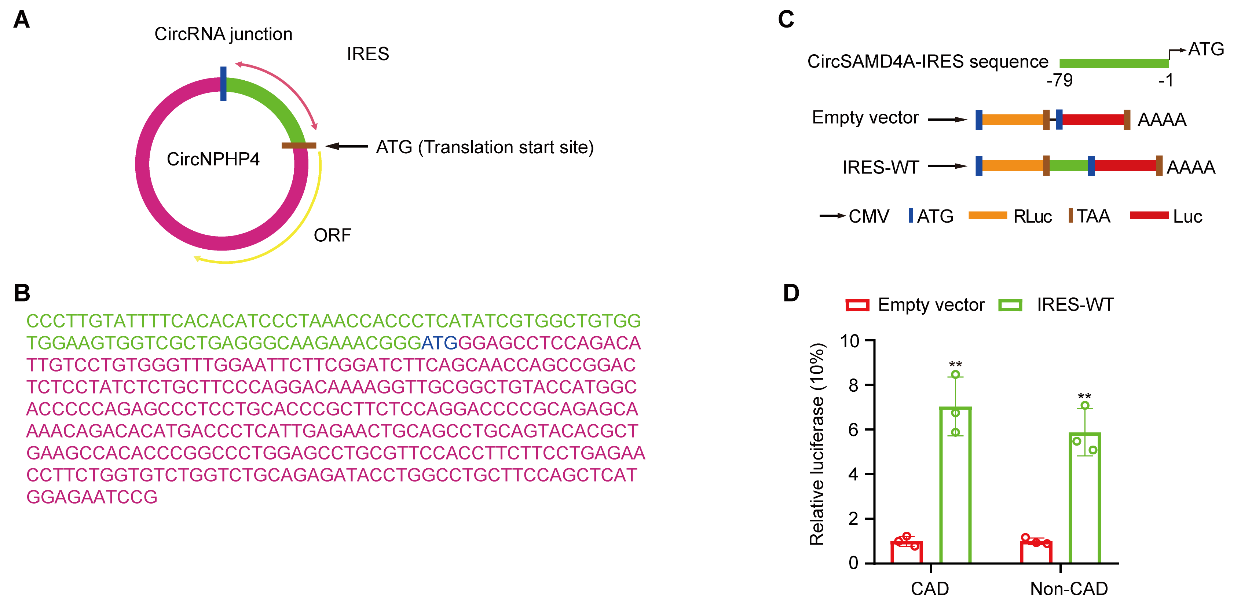


Supplementary Fig. S9. Evaluation of the coding ability of circNPHP4. (A) The putative open reading frame (ORF) in circNPHP4 (B) The sequences of the putative ORF are shown in blue, internal ribosomal entrance site (IRES) sequences are shown in green. (C) IRES sequences in circNPHP4 were cloned between Rluc and Luc reporter genes with independent start and stop codons. (D) The relative luciferase activity of Luc/ Rluc in the above vectors was tested. Data are presented as means ± SD; significant difference was identified with Student's t test. *P < 0.05; **P < 0.01; ns (not significant).


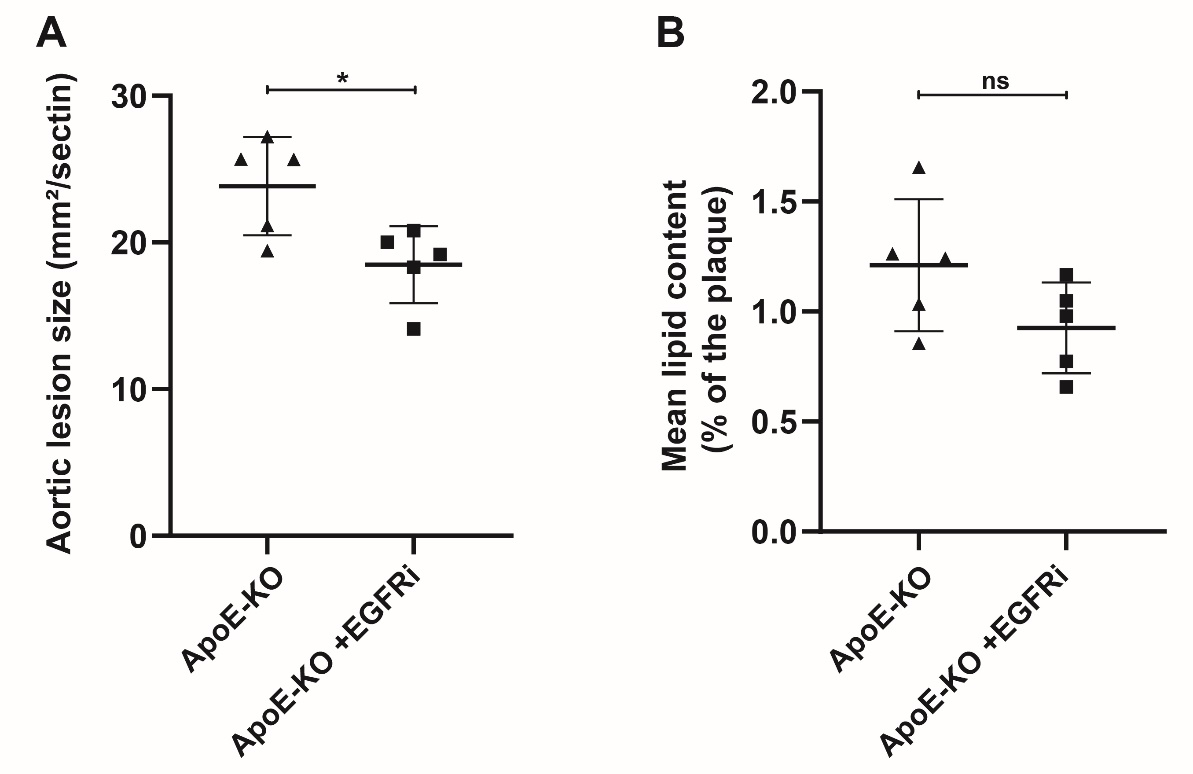


Supplementary Fig. S10. Gefitinib partly attenuated severity of CAD in HFD-treated Apo KO mice. (A) Quantification of aortic lesion size by H&E-staining on aortic sinus. (B) Assessment of lipid content by Oil-red-O-staining on aortic sinus. Data are presented as means ± SD; significant difference was identified with Student's t test. *P < 0.05; **P < 0.01; ns (not significant). N=5.

**Supplementary tables:**

Supplementary Table 2 Primers and probes used in the study

| Gene | | Sequence | Product length  （bp） |
| --- | --- | --- | --- |
| hsa_circ_0009135 | | 5’- CGTTCCACCTTCTTCCTGAG-3’ | 124 |
|  |  | 3’- CCACGATATGAGGGTGGTTT-5’ |  |
| hsa_circ_0063332 | | 5’- AGACGTTGGCGTATCTCCTG-3’ | 149 |
|  |  | 3’- GGTTTAGGCGTCTCCTTCCT-5’ |  |
| hsa_circ_0000375 | | 5’- CCTGACTTGGGAGGAGACTG -3’ | 160 |
|  |  | 3’- ACTCTCCGACAGGGAGGTGT-5’ |  |
| hsa_circ_0063331 | | 5’- CAGACTGGCTCTGGGAAGAC -3’ | 192 |
|  |  | 3’- TTGGGGAGCTCACTCAAATC -5’ |  |
| hsa_circ_0088152 | | 5’- AACACACACACGTGCACACA-3’ | 146 |
|  |  | 3’- ATCTGGTCCTGGCTTGACCT -5’ |  |
| hsa_circ_0048131 | | 5’- AGGAGGAGGAAGAGCTGACC-3’ | 127 |
|  |  | 3’- ACATGCTCCAGGCAGTTGAT-5’ |  |
| hsa_circ_0070762 | | 5’- GGACTCACCTGTTATCGATGG -3’ | 125 |
|  |  | 3’- GTTTTGTTCCCTTTGGTTTT -5’ |  |
| hsa_circ_0070761 | | 5’- CAGCCTGGAAGTATGCTATCAC -3’ | 126 |
|  |  | 3’- GGACGAAGGGTCTTTGCTTA -5’ |  |
| hsa_circ_0026102 | | 5’- CACCTGTGCTCTATGCCAAC -3’ | 151 |
|  |  | 3’- CCAATCAAATGGCAACTCAA -5’ |  |
| hsa_circ_0048121 | | 5’- CACCGCCTTCTACATCATCC -3’ | 131 |
|  |  | 3’- ATGTGGATGTAGGCCAGGAC -5’ |  |
| ICAM-1 | | 5’- ACATCTGTGTCCCCCTCAAA -3’ | 132 |
|  |  | 3’- AGGCAGGAGCAACTCCTTTT -5’ |  |
| VCAM-1  GAPDH | | 5’- AAGATGGTCGTGATCCTTGG -3’ | 142  150 |
|  |  | 3’- CTGTGGTGCTGCAAGTCAAT -5’  3’- GAAAGCCTGCCGGTGACTAA -5’  3’- GCCCAATACGACCAAATCAGAG -5’ |  |
|  |  |  |  |
| CircRNA probes | | | |
| CircNHPH4 | 5’-CCAGCTCATGGAGAATCCGCCCTTGTATTTTCACACATCCCTAA -3’-biotin | |  |
| Control | 5’-CTAAGACTAGGTGTTCGGAGGGAAAACAAAAAGAGATATCAGAA -3’-biotin | |  |
| miRNA probes | | | |
| hsa-miR-1205 | biotin-5’- TCTGCAGGGTTTGCTTTGAG -3’-biotin | |  |
| hsa-miR-1231 | biotin-5’- GTGTCTGGGCGGACAGCTGC -3’-biotin | |  |
| hsa-miR-136 | biotin-5’- ACTCCATTTGTTTTGATGATGGA -3’-biotin | |  |
| hsa-miR-140-3p | biotin-5’- CAGTGGTTTTACCCTATGGTAG -3’-biotin | |  |
| hsa-miR-217 | biotin-5’- TACTGCATCAGGAACTGATTGGA -3’-biotin | |  |
| hsa-miR-330-5p | biotin-5’- TCTCTGGGCCTGTGTCTTAGGC -3’-biotin | |  |
| hsa-miR-515-5p | biotin-5’- TTCTCCAAAAGAAAGCACTTTCTG -3’-biotin | |  |
| hsa-miR-516b | biotin-5’- ATCTGGAGGTAAGAAGCACTTT -3’-biotin | |  |
| hsa-miR-556-5p | biotin-5’- GATGAGCTCATTGTAATATGAG -3’-biotin | |  |
| hsa-miR-558 | biotin-5’- GATGAGCTCATTGTAATATGAG -3’-biotin | |  |
| FISH probes | | | |
| CircNHPH4 | digoxin-5’-CAGCTCATGGAGAATCCGCCCTTGTATTTTCACACATCCCTAA-3’-digoxin | |  |
| Negative Control | digoxin-5’-TAAGACTAGGTGTTCGGAGGGAAAACAAAAAGAGATATCAGAA -3’- digoxin | |  |
| siRNAs |  | |  |
| CircNHPH4 | 5’- CCTCTTAGGCGGGAACATAAA -3’ | |  |

Supplementary Table 3 Differential expression of m**onocyte-derived exosomal circRNAs** between the CAD and control samples using microarray detection

| **Probe ID** | **T test p-value** | **Fold Change** | | **Normalized signal (log2) of CAD** | | | **Normalized signal (log2) of control** | | |
| --- | --- | --- | --- | --- | --- | --- | --- | --- | --- |
| **CircRNA Name** | **P values** | **Foldchange** | **Regulation** | **CAD1** | **CAD2** | **CAD3** | **Control1** | **Control2** | **Control3** |
| hsa_circ_0022374 | 0.0000 | 0.1340 | down | 0.9190 | 0.8372 | 0.8572 | 3.8361 | 3.7223 | 3.7531 |
| hsa_circ_0049123 | 0.0096 | 0.1085 | down | 7.9237 | 7.9440 | 6.4222 | 10.3033 | 10.0416 | 11.5421 |
| hsa_circ_0074365 | 0.0019 | 0.1100 | down | 0.6836 | 1.5753 | 0.7014 | 3.8815 | 4.4461 | 4.3187 |
| hsa_circ_0083981 | 0.0008 | 0.1350 | down | 1.9125 | 1.4583 | 2.2700 | 4.7123 | 4.4379 | 5.1734 |
| hsa_circ_0020553 | 0.0005 | 0.1405 | down | 0.7123 | 0.6945 | 0.7388 | 3.3990 | 3.6466 | 3.5816 |
| hsa_circ_0060912 | 0.0069 | 0.1422 | down | 0.8703 | 0.8443 | 0.7964 | 3.9578 | 3.7268 | 3.1558 |
| hsa_circ_0030980 | 0.0065 | 0.1427 | down | 1.6888 | 0.6831 | 0.6985 | 3.6466 | 3.9780 | 4.0823 |
| hsa_circ_0047467 | 0.0041 | 0.1446 | down | 0.7242 | 1.6501 | 0.7286 | 3.5810 | 4.0862 | 3.9668 |
| hsa_circ_0050855 | 0.0057 | 0.1532 | down | 0.7469 | 0.7298 | 1.8182 | 3.1878 | 3.8682 | 4.3985 |
| hsa_circ_0006958 | 0.0079 | 0.1548 | down | 1.0606 | 0.9452 | 0.9574 | 3.1413 | 3.9533 | 3.8229 |
| hsa_circ_0039114 | 0.0030 | 0.1600 | down | 0.8117 | 0.8610 | 0.8864 | 3.1709 | 3.6272 | 3.6455 |
| hsa_circ_0048787 | 0.0043 | 0.1636 | down | 0.9360 | 0.9787 | 1.8850 | 3.7298 | 3.7893 | 4.2650 |
| hsa_circ_0072757 | 0.0004 | 0.1667 | down | 0.8337 | 0.8066 | 0.8663 | 3.3200 | 3.5591 | 3.3698 |
| hsa_circ_0032475 | 0.0004 | 0.1732 | down | 0.8586 | 0.7730 | 1.0140 | 3.1522 | 3.5906 | 3.4661 |
| hsa_circ_0067208 | 0.0007 | 0.1745 | down | 8.6759 | 9.3473 | 9.1151 | 11.3144 | 11.8183 | 11.5965 |
| hsa_circ_0009364 | 0.0041 | 0.1750 | down | 0.8912 | 0.9742 | 0.8307 | 3.1403 | 3.2695 | 3.7585 |
| hsa_circ_0069374 | 0.0022 | 0.1751 | down | 7.7866 | 8.6619 | 8.4835 | 10.4481 | 11.1258 | 10.9570 |
| hsa_circ_0011030 | 0.0012 | 0.1751 | down | 1.3497 | 1.0296 | 1.0501 | 3.3801 | 4.0099 | 3.5254 |
| hsa_circ_0007409 | 0.0002 | 0.1759 | down | 0.8306 | 0.7966 | 1.1951 | 3.3359 | 3.3042 | 3.7041 |
| hsa_circ_0069449 | 0.0037 | 0.1773 | down | 1.8303 | 0.7907 | 1.3665 | 3.5556 | 3.8046 | 4.2184 |
| hsa_circ_0043368 | 0.0067 | 0.1825 | down | 1.7039 | 0.6870 | 1.7914 | 3.5906 | 3.7505 | 4.3322 |
| hsa_circ_0039054 | 0.0029 | 0.1843 | down | 0.8125 | 1.7788 | 1.1784 | 3.2548 | 4.0193 | 3.8751 |
| hsa_circ_0045674 | 0.0021 | 0.1848 | down | 0.8177 | 1.2624 | 1.6161 | 3.1636 | 3.8454 | 3.9798 |
| hsa_circ_0007960 | 0.0036 | 0.1876 | down | 0.9616 | 0.8909 | 1.2026 | 2.9594 | 3.5798 | 3.6787 |
| hsa_circ_0061868 | 0.0028 | 0.1900 | down | 0.7848 | 0.7762 | 0.7739 | 3.0444 | 3.4159 | 3.0292 |
| hsa_circ_0043898 | 0.0058 | 0.1912 | down | 1.8138 | 0.8552 | 0.8833 | 3.8068 | 3.2760 | 3.7877 |
| hsa_circ_0019252 | 0.0055 | 0.1913 | down | 0.8228 | 0.7941 | 1.7448 | 2.9914 | 3.4837 | 4.0616 |
| hsa_circ_0060911 | 0.0006 | 0.1920 | down | 1.7734 | 1.1977 | 1.2884 | 3.6712 | 4.0948 | 3.6608 |
| hsa_circ_0074517 | 0.0060 | 0.1950 | down | 0.7976 | 1.0722 | 1.8158 | 3.4188 | 3.9398 | 3.5494 |
| hsa_circ_0042282 | 0.0021 | 0.1960 | down | 7.7510 | 8.5586 | 8.3980 | 10.2034 | 10.9121 | 10.6768 |
| hsa_circ_0046489 | 0.0096 | 0.1965 | down | 1.4722 | 2.2911 | 1.2654 | 3.8788 | 4.2686 | 4.1115 |
| hsa_circ_0044467 | 0.0036 | 0.1982 | down | 1.4099 | 2.3142 | 2.0966 | 3.9338 | 4.0894 | 4.7959 |
| hsa_circ_0044547 | 0.0021 | 0.1998 | down | 6.5514 | 7.1057 | 6.9778 | 8.9108 | 8.9734 | 9.6538 |
| hsa_circ_0039591 | 0.0001 | 0.2020 | down | 0.9860 | 0.9165 | 0.9917 | 3.2174 | 3.3847 | 3.2090 |
| hsa_circ_0042281 | 0.0035 | 0.2062 | down | 7.6272 | 8.5171 | 8.3839 | 10.1447 | 10.9050 | 10.3506 |
| hsa_circ_0063824 | 0.0026 | 0.2090 | down | 9.1529 | 8.2980 | 8.5652 | 10.5612 | 11.2824 | 10.9945 |
| hsa_circ_0000809 | 0.0023 | 0.2091 | down | 0.9643 | 1.0033 | 1.6771 | 3.2377 | 3.7357 | 3.5182 |
| hsa_circ_0044526 | 0.0077 | 0.2124 | down | 6.9729 | 6.9619 | 6.9599 | 8.8993 | 9.0684 | 9.5507 |
| hsa_circ_0082425 | 0.0031 | 0.2141 | down | 9.2345 | 8.3722 | 8.5855 | 10.7043 | 11.3264 | 10.9050 |
| hsa_circ_0063823 | 0.0021 | 0.2152 | down | 9.0498 | 8.2868 | 8.5430 | 10.4883 | 11.2376 | 10.8115 |
| hsa_circ_0004254 | 0.0075 | 0.2154 | down | 1.4864 | 1.6486 | 0.7826 | 3.4450 | 3.7525 | 3.4820 |
| hsa_circ_0051336 | 0.0063 | 0.2168 | down | 0.7107 | 0.7098 | 1.4598 | 3.0334 | 3.3564 | 3.2244 |
| hsa_circ_0066181 | 0.0097 | 0.2216 | down | 12.0714 | 11.1258 | 11.0741 | 13.5027 | 13.9629 | 13.5093 |
| hsa_circ_0081129 | 0.0003 | 0.2239 | down | 1.2182 | 1.4040 | 1.4738 | 3.4859 | 3.5083 | 3.5898 |
| hsa_circ_0085710 | 0.0083 | 0.2245 | down | 8.9365 | 8.2629 | 8.6759 | 10.2146 | 10.6768 | 11.3144 |
| hsa_circ_0036040 | 0.0097 | 0.2255 | down | 3.0162 | 1.9167 | 2.0089 | 4.2067 | 4.1885 | 5.0773 |
| hsa_circ_0043845 | 0.0088 | 0.2268 | down | 0.8456 | 1.8349 | 1.4974 | 3.3587 | 3.6059 | 3.7731 |
| hsa_circ_0085717 | 0.0078 | 0.2313 | down | 8.8665 | 8.3078 | 8.6714 | 10.1977 | 10.6360 | 11.2201 |
| hsa_circ_0071731 | 0.0045 | 0.2318 | down | 2.9430 | 3.7346 | 3.8021 | 5.2190 | 5.8781 | 5.7739 |
| hsa_circ_0026389 | 0.0013 | 0.2333 | down | 5.0168 | 4.3777 | 4.5398 | 6.4145 | 6.9330 | 6.9084 |
| hsa_circ_0044534 | 0.0024 | 0.2383 | down | 7.4061 | 7.7298 | 7.4375 | 9.2767 | 9.5090 | 9.9387 |
| hsa_circ_0077141 | 0.0094 | 0.2412 | down | 1.8685 | 0.7583 | 1.5953 | 3.0510 | 3.7260 | 3.7181 |
| hsa_circ_0090602 | 0.0013 | 0.2427 | down | 2.2006 | 1.5865 | 2.0069 | 3.7370 | 4.0879 | 4.1322 |
| hsa_circ_0064249 | 0.0005 | 0.2521 | down | 2.1811 | 2.6414 | 2.4670 | 4.3985 | 4.5977 | 4.2748 |
| hsa_circ_0088924 | 0.0039 | 0.2536 | down | 1.7722 | 1.9121 | 2.0254 | 3.5151 | 4.1440 | 3.9311 |
| hsa_circ_0090913 | 0.0037 | 0.2546 | down | 0.7402 | 1.3146 | 1.5305 | 3.0549 | 3.4916 | 3.0217 |
| hsa_circ_0016390 | 0.0082 | 0.2562 | down | 1.5586 | 1.4002 | 1.6306 | 3.0883 | 3.4468 | 3.8557 |
| hsa_circ_0021956 | 0.0055 | 0.2575 | down | 0.7629 | 1.4202 | 1.5761 | 3.0612 | 3.5015 | 3.1523 |
| hsa_circ_0051958 | 0.0073 | 0.2613 | down | 0.8575 | 1.8181 | 1.4775 | 3.0908 | 3.6615 | 3.3095 |
| hsa_circ_0086132 | 0.0094 | 0.2688 | down | 2.4947 | 1.4193 | 1.9494 | 4.2356 | 3.4188 | 3.9774 |
| hsa_circ_0015609 | 0.0067 | 0.2703 | down | 11.9893 | 11.8378 | 11.1356 | 13.3923 | 13.8683 | 13.4512 |
| hsa_circ_0041615 | 0.0024 | 0.2747 | down | 2.1513 | 1.8671 | 1.4719 | 3.4766 | 3.9330 | 3.7167 |
| hsa_circ_0024382 | 0.0007 | 0.2766 | down | 1.7537 | 1.6970 | 1.3334 | 3.2847 | 3.3209 | 3.7324 |
| hsa_circ_0012867 | 0.0093 | 0.2811 | down | 2.8474 | 3.0759 | 2.8449 | 4.2744 | 4.9683 | 4.9314 |
| hsa_circ_0083837 | 0.0055 | 0.2834 | down | 2.7516 | 2.2204 | 2.7746 | 4.3913 | 4.3373 | 4.5336 |
| hsa_circ_0076218 | 0.0001 | 0.2843 | down | 2.3266 | 2.3660 | 2.6029 | 4.1864 | 4.4004 | 4.1556 |
| hsa_circ_0058676 | 0.0079 | 0.2844 | down | 2.9476 | 2.1372 | 2.1378 | 3.9143 | 4.0476 | 4.7260 |
| hsa_circ_0017350 | 0.0099 | 0.2867 | down | 3.7066 | 2.9729 | 3.5369 | 5.1127 | 5.2847 | 5.3149 |
| hsa_circ_0051234 | 0.0011 | 0.2904 | down | 3.0698 | 2.5714 | 2.6257 | 4.3773 | 4.7780 | 4.4866 |
| hsa_circ_0034398 | 0.0014 | 0.2963 | down | 1.3805 | 1.7584 | 1.5155 | 2.9876 | 3.3846 | 3.5204 |
| hsa_circ_0059617 | 0.0058 | 0.2984 | down | 2.6782 | 2.2307 | 2.9345 | 4.2292 | 4.5449 | 4.3717 |
| hsa_circ_0022411 | 0.0066 | 0.3044 | down | 3.4096 | 2.8822 | 2.5431 | 4.5152 | 4.4675 | 5.0564 |
| hsa_circ_0050648 | 0.0012 | 0.3072 | down | 2.3002 | 2.1216 | 2.2183 | 4.0455 | 3.6865 | 3.9961 |
| hsa_circ_0008768 | 0.0037 | 0.3082 | down | 1.9035 | 1.2141 | 1.3637 | 2.9301 | 3.2395 | 3.4530 |
| hsa_circ_0009912 | 0.0068 | 0.3104 | down | 1.8227 | 1.0142 | 1.6843 | 2.8506 | 3.5018 | 3.2798 |
| hsa_circ_0058014 | 0.0034 | 0.3115 | down | 2.9863 | 3.1151 | 3.6505 | 4.6321 | 5.1450 | 5.0612 |
| hsa_circ_0055300 | 0.0065 | 0.3119 | down | 2.4286 | 1.8634 | 2.0614 | 3.6218 | 4.2445 | 3.4652 |
| hsa_circ_0081652 | 0.0007 | 0.3143 | down | 2.1343 | 1.8391 | 2.0488 | 3.8966 | 3.4618 | 3.6561 |
| hsa_circ_0050864 | 0.0002 | 0.3143 | down | 1.8823 | 1.9437 | 1.6701 | 3.3146 | 3.5478 | 3.6383 |
| hsa_circ_0038762 | 0.0015 | 0.3160 | down | 1.6971 | 1.7805 | 1.7741 | 3.2280 | 3.4814 | 3.5127 |
| hsa_circ_0020325 | 0.0080 | 0.3165 | down | 9.0068 | 8.3667 | 8.3742 | 10.1602 | 10.4374 | 10.2113 |
| hsa_circ_0066180 | 0.0064 | 0.3196 | down | 12.1403 | 12.0714 | 12.1828 | 13.5897 | 14.0607 | 13.6353 |
| hsa_circ_0015858 | 0.0051 | 0.3215 | down | 1.6996 | 1.6542 | 2.1449 | 3.0921 | 3.4256 | 3.8454 |
| hsa_circ_0041568 | 0.0008 | 0.3218 | down | 2.3574 | 2.2973 | 1.9388 | 3.9231 | 3.6374 | 3.9538 |
| hsa_circ_0057507 | 0.0044 | 0.3221 | down | 1.7437 | 2.4542 | 1.9717 | 3.5484 | 3.9913 | 3.5836 |
| hsa_circ_0054853 | 0.0017 | 0.3222 | down | 4.1325 | 4.0813 | 4.5878 | 5.6983 | 6.1157 | 5.9151 |
| hsa_circ_0001334 | 0.0017 | 0.3244 | down | 2.9686 | 2.7425 | 3.2946 | 4.3893 | 4.7903 | 4.7220 |
| hsa_circ_0067475 | 0.0048 | 0.3263 | down | 1.8072 | 1.5297 | 2.2143 | 3.8182 | 3.4686 | 3.1079 |
| hsa_circ_0080739 | 0.0046 | 0.3305 | down | 3.9716 | 3.3842 | 3.3131 | 4.8868 | 5.2507 | 5.3742 |
| hsa_circ_0042174 | 0.0022 | 0.3311 | down | 3.3152 | 3.3044 | 3.0140 | 4.5765 | 5.1000 | 4.7081 |
| hsa_circ_0058177 | 0.0056 | 0.3331 | down | 3.3945 | 2.8183 | 3.5447 | 4.5290 | 4.9493 | 5.0787 |
| hsa_circ_0040029 | 0.0004 | 0.3334 | down | 2.0434 | 2.2708 | 2.0917 | 3.6659 | 3.7760 | 3.7254 |
| hsa_circ_0008685 | 0.0010 | 0.3359 | down | 6.1285 | 5.8606 | 6.1971 | 7.4018 | 7.8646 | 7.6259 |
| hsa_circ_0005806 | 0.0057 | 0.3365 | down | 8.9946 | 8.5222 | 8.7623 | 10.3834 | 10.2907 | 10.3559 |
| hsa_circ_0091809 | 0.0015 | 0.3422 | down | 2.7249 | 2.4324 | 2.9303 | 4.0589 | 4.4110 | 4.2808 |
| hsa_circ_0060958 | 0.0007 | 0.3431 | down | 2.3580 | 2.6716 | 2.7526 | 4.1084 | 4.3293 | 3.9818 |
| hsa_circ_0010867 | 0.0014 | 0.3472 | down | 9.0939 | 8.5962 | 8.8665 | 10.3107 | 10.2406 | 10.6011 |
| hsa_circ_0048426 | 0.0035 | 0.3499 | down | 3.5454 | 3.0436 | 3.1508 | 4.6368 | 4.8758 | 4.8117 |
| hsa_circ_0066978 | 0.0089 | 0.3510 | down | 2.5292 | 2.3780 | 1.9676 | 3.6876 | 3.4269 | 4.2294 |
| hsa_circ_0089782 | 0.0031 | 0.3553 | down | 3.5308 | 3.2323 | 3.8153 | 4.7790 | 5.3289 | 4.9514 |
| hsa_circ_0020492 | 0.0071 | 0.3555 | down | 4.9560 | 4.1938 | 4.6508 | 5.8178 | 6.3193 | 6.1940 |
| hsa_circ_0071651 | 0.0090 | 0.3561 | down | 9.2194 | 8.3959 | 8.6920 | 10.3727 | 9.9387 | 10.5256 |
| hsa_circ_0016544 | 0.0040 | 0.3566 | down | 2.4937 | 2.2932 | 1.8618 | 3.5636 | 3.9805 | 3.5998 |
| hsa_circ_0009130 | 0.0042 | 0.3569 | down | 6.0181 | 5.4425 | 5.9762 | 7.0437 | 7.6223 | 7.2373 |
| hsa_circ_0084491 | 0.0073 | 0.3573 | down | 2.9091 | 2.5234 | 2.1638 | 3.9934 | 3.8426 | 4.2764 |
| hsa_circ_0058973 | 0.0043 | 0.3593 | down | 3.2303 | 3.5919 | 3.4942 | 4.5550 | 5.1428 | 5.0093 |
| hsa_circ_0085405 | 0.0005 | 0.3603 | down | 2.4554 | 2.3810 | 2.4209 | 3.9514 | 3.7715 | 3.9464 |
| hsa_circ_0076296 | 0.0031 | 0.3607 | down | 8.7764 | 8.2186 | 8.4450 | 9.7839 | 10.1238 | 9.9804 |
| hsa_circ_0089670 | 0.0008 | 0.3655 | down | 2.1414 | 2.1872 | 2.0959 | 3.5953 | 3.7109 | 3.4658 |
| hsa_circ_0088874 | 0.0013 | 0.3669 | down | 1.8367 | 2.1438 | 1.8137 | 3.2624 | 3.2177 | 3.6394 |
| hsa_circ_0020323 | 0.0013 | 0.3678 | down | 3.9483 | 3.6479 | 4.0862 | 5.1657 | 5.4046 | 5.4591 |
| hsa_circ_0058958 | 0.0038 | 0.3690 | down | 1.7506 | 1.7763 | 1.6750 | 2.9982 | 3.3765 | 3.1176 |
| hsa_circ_0004291 | 0.0027 | 0.3695 | down | 6.0153 | 5.5865 | 6.1136 | 7.0976 | 7.4752 | 7.4729 |
| hsa_circ_0014875 | 0.0050 | 0.3719 | down | 2.1713 | 2.5760 | 2.5656 | 3.9308 | 3.9009 | 3.7947 |
| hsa_circ_0025418 | 0.0095 | 0.3740 | down | 2.0595 | 2.6852 | 2.7524 | 4.0495 | 4.0761 | 3.6934 |
| hsa_circ_0052271 | 0.0072 | 0.3741 | down | 4.1154 | 3.5647 | 3.9710 | 4.9828 | 5.1944 | 5.6909 |
| hsa_circ_0020488 | 0.0098 | 0.3741 | down | 4.5911 | 4.5865 | 4.8634 | 5.6940 | 6.3733 | 6.1658 |
| hsa_circ_0053054 | 0.0045 | 0.3762 | down | 3.0656 | 2.4874 | 3.0084 | 4.2679 | 4.5312 | 4.0143 |
| hsa_circ_0040695 | 0.0005 | 0.3778 | down | 4.2349 | 3.9215 | 4.1877 | 5.4773 | 5.7042 | 5.3748 |
| hsa_circ_0007571 | 0.0047 | 0.3785 | down | 4.0891 | 4.0170 | 4.5040 | 5.2447 | 5.6973 | 5.8550 |
| hsa_circ_0008182 | 0.0011 | 0.3786 | down | 2.1642 | 2.0334 | 2.3858 | 3.5133 | 3.5849 | 3.7046 |
| hsa_circ_0081645 | 0.0031 | 0.3787 | down | 2.1529 | 2.3562 | 2.2695 | 3.3969 | 3.7298 | 3.8274 |
| hsa_circ_0037079 | 0.0008 | 0.3799 | down | 3.1467 | 2.9108 | 2.8338 | 4.4298 | 4.3998 | 4.2641 |
| hsa_circ_0003880 | 0.0033 | 0.3800 | down | 2.8509 | 3.3282 | 2.8918 | 4.5475 | 4.6092 | 4.1009 |
| hsa_circ_0077998 | 0.0092 | 0.3807 | down | 3.9230 | 3.6022 | 3.6115 | 5.0150 | 4.7722 | 5.4653 |
| hsa_circ_0009782 | 0.0050 | 0.3813 | down | 2.6317 | 2.3817 | 3.0195 | 4.0952 | 4.2952 | 3.8541 |
| hsa_circ_0000948 | 0.0081 | 0.3817 | down | 2.3504 | 1.7845 | 2.1426 | 3.5856 | 3.5128 | 3.3967 |
| hsa_circ_0013464 | 0.0032 | 0.3822 | down | 3.9019 | 3.5729 | 3.4285 | 5.0936 | 5.2548 | 4.7054 |
| hsa_circ_0050147 | 0.0082 | 0.3825 | down | 2.0366 | 1.6979 | 1.9534 | 2.9283 | 3.6020 | 3.2603 |
| hsa_circ_0021587 | 0.0010 | 0.3854 | down | 2.1522 | 2.2651 | 1.9633 | 3.3707 | 3.7308 | 3.3928 |
| hsa_circ_0049088 | 0.0012 | 0.3862 | down | 3.4367 | 3.2673 | 3.0088 | 4.4767 | 4.5640 | 4.8019 |
| hsa_circ_0059251 | 0.0017 | 0.3871 | down | 3.8028 | 3.5172 | 3.4100 | 4.8903 | 5.1972 | 4.7410 |
| hsa_circ_0037247 | 0.0082 | 0.3901 | down | 1.6945 | 1.9579 | 2.3487 | 2.9599 | 3.4889 | 3.6213 |
| hsa_circ_0064559 | 0.0094 | 0.3914 | down | 3.3612 | 3.1818 | 3.7168 | 4.7344 | 4.8681 | 4.7656 |
| hsa_circ_0076295 | 0.0045 | 0.3915 | down | 8.8487 | 8.2663 | 8.4571 | 9.6830 | 10.0547 | 9.9308 |
| hsa_circ_0080413 | 0.0089 | 0.3933 | down | 3.9424 | 3.3632 | 3.2792 | 4.7575 | 5.1394 | 4.7895 |
| hsa_circ_0074356 | 0.0069 | 0.3974 | down | 3.1349 | 3.0052 | 3.6464 | 4.5006 | 4.8719 | 4.4528 |
| hsa_circ_0038720 | 0.0017 | 0.3983 | down | 3.6154 | 3.2358 | 3.2661 | 4.6859 | 4.8452 | 4.5900 |
| hsa_circ_0000453 | 0.0015 | 0.4005 | down | 8.9295 | 8.5399 | 8.8041 | 9.9474 | 10.1316 | 10.1725 |
| hsa_circ_0029225 | 0.0068 | 0.4036 | down | 2.4574 | 2.8188 | 2.1497 | 3.5046 | 3.9397 | 3.9435 |
| hsa_circ_0058892 | 0.0003 | 0.4045 | down | 2.8925 | 2.9996 | 2.7271 | 4.1164 | 4.3366 | 4.0830 |
| hsa_circ_0025133 | 0.0058 | 0.4054 | down | 3.3417 | 2.7289 | 3.1709 | 4.4231 | 4.6041 | 4.1540 |
| hsa_circ_0051040 | 0.0095 | 0.4063 | down | 1.9187 | 2.2316 | 2.0744 | 2.9959 | 3.6181 | 3.4557 |
| hsa_circ_0048562 | 0.0079 | 0.4093 | down | 2.7254 | 3.1515 | 3.4096 | 4.5224 | 4.5722 | 4.0922 |
| hsa_circ_0010587 | 0.0055 | 0.4107 | down | 8.8168 | 8.2220 | 8.4012 | 9.4666 | 9.9308 | 9.9141 |
| hsa_circ_0009200 | 0.0085 | 0.4143 | down | 3.1224 | 2.4700 | 2.9094 | 4.0227 | 4.4519 | 3.8470 |
| hsa_circ_0065644 | 0.0047 | 0.4146 | down | 5.8485 | 5.2654 | 5.5452 | 6.5852 | 6.9599 | 6.9527 |
| hsa_circ_0036677 | 0.0087 | 0.4156 | down | 10.0289 | 9.4720 | 9.6417 | 11.1224 | 10.9570 | 10.9121 |
| hsa_circ_0068043 | 0.0084 | 0.4158 | down | 2.6324 | 2.1764 | 2.1012 | 3.5072 | 3.7213 | 3.5289 |
| hsa_circ_0060960 | 0.0027 | 0.4162 | down | 3.2588 | 2.8251 | 2.8436 | 4.2017 | 4.4396 | 4.1023 |
| hsa_circ_0001871 | 0.0042 | 0.4179 | down | 5.9095 | 5.6026 | 5.5649 | 6.6288 | 7.0936 | 7.1067 |
| hsa_circ_0039679 | 0.0096 | 0.4203 | down | 3.7685 | 3.2256 | 3.5204 | 4.6775 | 4.8472 | 4.7867 |
| hsa_circ_0050572 | 0.0012 | 0.4203 | down | 2.7411 | 2.6430 | 3.0085 | 4.0753 | 3.8583 | 4.2133 |
| hsa_circ_0073012 | 0.0017 | 0.4225 | down | 4.4767 | 4.0943 | 4.3180 | 5.2968 | 5.6853 | 5.6314 |
| hsa_circ_0023105 | 0.0026 | 0.4238 | down | 3.2757 | 3.2334 | 3.4305 | 4.3709 | 4.4966 | 4.7667 |
| hsa_circ_0026567 | 0.0088 | 0.4292 | down | 2.6140 | 2.4374 | 2.0290 | 3.7504 | 3.4703 | 3.5673 |
| hsa_circ_0059708 | 0.0021 | 0.4336 | down | 4.8596 | 5.1850 | 4.8941 | 5.9287 | 6.2948 | 6.3219 |
| hsa_circ_0045716 | 0.0054 | 0.4367 | down | 1.9538 | 1.5626 | 2.1131 | 2.8749 | 3.3413 | 3.0127 |
| hsa_circ_0043328 | 0.0029 | 0.4372 | down | 2.5931 | 2.3724 | 2.2669 | 3.5828 | 3.8396 | 3.3723 |
| hsa_circ_0082906 | 0.0060 | 0.4389 | down | 2.3818 | 2.3637 | 2.6852 | 3.3943 | 3.9525 | 3.6163 |
| hsa_circ_0076032 | 0.0021 | 0.4390 | down | 2.4761 | 2.1603 | 2.4698 | 3.7285 | 3.3124 | 3.6191 |
| hsa_circ_0078057 | 0.0021 | 0.4392 | down | 2.0686 | 1.9440 | 2.3069 | 3.3965 | 3.2039 | 3.2972 |
| hsa_circ_0026175 | 0.0099 | 0.4422 | down | 4.0806 | 3.6515 | 3.7208 | 4.7030 | 4.8961 | 5.3457 |
| hsa_circ_0009216 | 0.0054 | 0.4425 | down | 4.7007 | 4.1639 | 4.3175 | 5.4476 | 5.8522 | 5.4240 |
| hsa_circ_0086020 | 0.0068 | 0.4430 | down | 10.1055 | 9.5119 | 9.7420 | 11.0741 | 11.1129 | 10.7293 |
| hsa_circ_0049358 | 0.0036 | 0.4434 | down | 8.0836 | 7.9309 | 8.2408 | 8.9778 | 9.4079 | 9.3684 |
| hsa_circ_0044159 | 0.0031 | 0.4450 | down | 3.4970 | 3.4837 | 3.4494 | 4.6689 | 4.7541 | 4.5001 |
| hsa_circ_0052392 | 0.0088 | 0.4491 | down | 2.5346 | 2.9270 | 3.1611 | 3.9915 | 4.2713 | 3.8616 |
| hsa_circ_0031225 | 0.0020 | 0.4494 | down | 2.7095 | 2.7641 | 2.4104 | 3.8134 | 3.9641 | 3.5650 |
| hsa_circ_0092107 | 0.0081 | 0.4505 | down | 1.7129 | 2.2473 | 2.2191 | 3.1102 | 3.4349 | 3.1209 |
| hsa_circ_0058390 | 0.0049 | 0.4531 | down | 2.5089 | 2.6558 | 3.0208 | 3.6245 | 3.9899 | 4.0147 |
| hsa_circ_0064076 | 0.0025 | 0.4539 | down | 9.6219 | 9.1955 | 9.4375 | 10.5817 | 10.4028 | 10.7043 |
| hsa_circ_0020521 | 0.0021 | 0.4550 | down | 9.3031 | 8.8924 | 9.1430 | 10.2053 | 10.1008 | 10.4481 |
| hsa_circ_0022353 | 0.0086 | 0.4572 | down | 8.5939 | 8.8821 | 8.8055 | 9.5754 | 9.9234 | 10.1316 |
| hsa_circ_0026929 | 0.0011 | 0.4599 | down | 3.3695 | 3.4339 | 3.2010 | 4.5259 | 4.5697 | 4.2614 |
| hsa_circ_0042170 | 0.0026 | 0.4610 | down | 3.7911 | 3.3707 | 3.5068 | 4.7494 | 4.4578 | 4.8200 |
| hsa_circ_0063608 | 0.0087 | 0.4619 | down | 4.4094 | 4.2246 | 4.7198 | 5.4780 | 5.6707 | 5.5854 |
| hsa_circ_0041675 | 0.0045 | 0.4646 | down | 3.8113 | 3.3420 | 3.4621 | 4.5075 | 4.9053 | 4.5262 |
| hsa_circ_0090686 | 0.0084 | 0.4652 | down | 2.7472 | 2.5729 | 3.1164 | 4.0634 | 3.7754 | 3.9494 |
| hsa_circ_0026484 | 0.0053 | 0.4655 | down | 3.3166 | 2.9550 | 2.8225 | 4.0695 | 4.3337 | 4.0272 |
| hsa_circ_0071821 | 0.0066 | 0.4692 | down | 8.7156 | 9.1487 | 9.1080 | 9.9834 | 10.0884 | 10.2053 |
| hsa_circ_0018443 | 0.0085 | 0.4711 | down | 2.7007 | 2.4247 | 3.0093 | 3.9527 | 3.5972 | 3.8781 |
| hsa_circ_0052512 | 0.0022 | 0.4758 | down | 4.1971 | 4.4503 | 4.5525 | 5.2567 | 5.6262 | 5.5299 |
| hsa_circ_0059746 | 0.0019 | 0.4766 | down | 2.4653 | 2.7778 | 2.5760 | 3.6836 | 3.8559 | 3.4805 |
| hsa_circ_0015038 | 0.0091 | 0.4769 | down | 4.4753 | 4.1134 | 4.6855 | 5.5452 | 5.6561 | 5.3138 |
| hsa_circ_0017890 | 0.0091 | 0.4782 | down | 3.9724 | 3.5020 | 3.6141 | 4.4535 | 5.0335 | 4.7793 |
| hsa_circ_0054874 | 0.0095 | 0.4786 | down | 2.3524 | 2.2735 | 2.4484 | 3.5735 | 3.5198 | 3.1375 |
| hsa_circ_0000055 | 0.0022 | 0.4789 | down | 4.6083 | 4.5529 | 4.8747 | 5.5168 | 5.8658 | 5.8358 |
| hsa_circ_0077584 | 0.0001 | 0.4810 | down | 8.9734 | 8.8458 | 8.9624 | 9.9141 | 9.9804 | 10.0547 |
| hsa_circ_0022695 | 0.0045 | 0.4816 | down | 2.7908 | 2.4519 | 2.7996 | 3.7621 | 3.6419 | 3.8214 |
| hsa_circ_0081398 | 0.0033 | 0.4823 | down | 3.5761 | 3.1861 | 3.4904 | 4.4832 | 4.3477 | 4.5956 |
| hsa_circ_0053131 | 0.0023 | 0.4871 | down | 4.6235 | 4.2952 | 4.3113 | 5.6354 | 5.2728 | 5.4366 |
| hsa_circ_0066798 | 0.0100 | 0.4926 | down | 2.7869 | 2.5034 | 2.7554 | 3.3873 | 3.7699 | 3.9186 |
| hsa_circ_0004377 | 0.0064 | 0.4931 | down | 5.2450 | 4.8771 | 5.3024 | 6.1264 | 6.3047 | 6.0794 |
| hsa_circ_0051697 | 0.0090 | 0.4943 | down | 4.2964 | 4.1280 | 3.7749 | 4.8972 | 5.3780 | 4.9743 |
| hsa_circ_0011131 | 0.0007 | 0.4952 | down | 3.4482 | 3.5633 | 3.6829 | 4.6341 | 4.5061 | 4.6023 |
| hsa_circ_0085925 | 0.0010 | 0.4968 | down | 2.6610 | 2.4827 | 2.3829 | 3.6249 | 3.3550 | 3.5740 |
| hsa_circ_0067138 | 0.0059 | 0.4969 | down | 2.7375 | 2.2646 | 2.6161 | 3.5627 | 3.7506 | 3.3445 |
| hsa_circ_0089341 | 0.0062 | 2.0168 | up | 6.1642 | 5.9653 | 5.7286 | 5.1980 | 4.7051 | 4.9087 |
| hsa_circ_0048949 | 0.0026 | 2.0252 | up | 4.5000 | 4.7175 | 4.3606 | 3.6488 | 3.2873 | 3.5852 |
| hsa_circ_0036010 | 0.0090 | 2.0367 | up | 7.5746 | 7.4419 | 7.4811 | 6.5943 | 6.5820 | 6.2144 |
| hsa_circ_0084020 | 0.0066 | 2.0396 | up | 4.1740 | 3.8137 | 3.7133 | 2.6944 | 3.1512 | 2.7693 |
| hsa_circ_0026106 | 0.0092 | 2.0601 | up | 5.4131 | 5.3807 | 5.7851 | 4.4271 | 4.5795 | 4.4758 |
| hsa_circ_0070157 | 0.0000 | 2.0642 | up | 3.8738 | 3.7711 | 3.7753 | 2.7925 | 2.6922 | 2.7987 |
| hsa_circ_0044409 | 0.0003 | 2.0671 | up | 5.2569 | 5.4387 | 5.2720 | 4.2672 | 4.1624 | 4.3929 |
| hsa_circ_0067708 | 0.0020 | 2.0732 | up | 4.6740 | 4.5405 | 4.8341 | 3.4762 | 3.5682 | 3.8388 |
| hsa_circ_0025045 | 0.0064 | 2.0801 | up | 4.0204 | 4.0850 | 3.6472 | 2.7512 | 2.8617 | 2.9967 |
| hsa_circ_0063226 | 0.0025 | 2.0838 | up | 5.8857 | 6.0290 | 6.1911 | 5.0052 | 5.0308 | 4.9053 |
| hsa_circ_0088710 | 0.0021 | 2.1087 | up | 4.7613 | 4.5895 | 4.4245 | 3.6164 | 3.5063 | 3.4375 |
| hsa_circ_0004721 | 0.0098 | 2.1416 | up | 5.2393 | 5.4975 | 5.8466 | 4.5503 | 4.5660 | 4.2083 |
| hsa_circ_0025195 | 0.0084 | 2.1419 | up | 4.2108 | 4.3009 | 4.4990 | 3.0515 | 3.0778 | 3.5449 |
| hsa_circ_0019728 | 0.0016 | 2.1518 | up | 4.0084 | 3.8540 | 4.2231 | 2.8108 | 2.8490 | 3.1138 |
| hsa_circ_0001402 | 0.0079 | 2.1637 | up | 6.8115 | 6.4128 | 6.9714 | 5.6350 | 5.8143 | 5.4371 |
| hsa_circ_0007486 | 0.0071 | 2.1700 | up | 6.4801 | 6.4185 | 6.9400 | 5.6724 | 5.5710 | 5.2704 |
| hsa_circ_0034401 | 0.0094 | 2.1810 | up | 4.7373 | 4.1474 | 4.3362 | 2.9733 | 3.5272 | 3.3551 |
| hsa_circ_0044011 | 0.0012 | 2.1836 | up | 4.6499 | 4.6043 | 4.6825 | 3.4476 | 3.6436 | 3.4581 |
| hsa_circ_0029161 | 0.0020 | 2.1842 | up | 5.5962 | 5.2990 | 5.6795 | 4.2837 | 4.5694 | 4.3518 |
| hsa_circ_0050882 | 0.0004 | 2.2232 | up | 5.6449 | 5.6923 | 5.4415 | 4.5695 | 4.3862 | 4.3685 |
| hsa_circ_0041653 | 0.0021 | 2.2513 | up | 4.5686 | 4.3464 | 4.5786 | 3.2927 | 3.1344 | 3.5371 |
| hsa_circ_0044012 | 0.0070 | 2.2637 | up | 5.1835 | 5.3391 | 5.6087 | 4.2686 | 4.2160 | 4.1401 |
| hsa_circ_0091109 | 0.0073 | 2.2818 | up | 4.5377 | 4.2539 | 4.3514 | 3.2795 | 3.3948 | 2.8596 |
| hsa_circ_0043336 | 0.0081 | 2.2930 | up | 4.0703 | 3.9867 | 4.4411 | 2.9490 | 2.9209 | 3.0736 |
| hsa_circ_0019301 | 0.0033 | 2.2983 | up | 4.2617 | 4.1389 | 4.1678 | 2.7961 | 3.0065 | 3.1456 |
| hsa_circ_0080791 | 0.0067 | 2.3051 | up | 4.9658 | 5.2728 | 5.5653 | 3.8422 | 4.2511 | 4.1280 |
| hsa_circ_0006924 | 0.0038 | 2.3134 | up | 4.7795 | 4.4295 | 4.6055 | 3.4599 | 3.5998 | 3.1013 |
| hsa_circ_0059104 | 0.0035 | 2.3147 | up | 5.2707 | 5.3310 | 5.6104 | 4.3917 | 4.2647 | 3.9030 |
| hsa_circ_0027395 | 0.0030 | 2.3226 | up | 7.7427 | 7.4087 | 7.6984 | 6.3675 | 6.4929 | 6.3607 |
| hsa_circ_0013543 | 0.0001 | 2.3228 | up | 5.9586 | 5.9875 | 6.0783 | 4.8143 | 4.8714 | 4.6878 |
| hsa_circ_0041222 | 0.0003 | 2.3731 | up | 5.2171 | 5.0550 | 5.3324 | 4.0884 | 3.8551 | 3.9240 |
| hsa_circ_0071464 | 0.0016 | 2.3838 | up | 5.5623 | 5.3041 | 5.1659 | 3.9951 | 4.0429 | 4.2501 |
| hsa_circ_0041654 | 0.0013 | 2.3977 | up | 3.9300 | 4.2651 | 4.2683 | 2.8581 | 2.7227 | 3.0977 |
| hsa_circ_0022866 | 0.0062 | 2.4030 | up | 6.5686 | 6.0071 | 6.1441 | 4.8162 | 5.1600 | 4.9896 |
| hsa_circ_0007755 | 0.0038 | 2.4264 | up | 4.6493 | 4.2363 | 4.4522 | 3.4661 | 3.0776 | 2.9333 |
| hsa_circ_0012074 | 0.0071 | 2.4304 | up | 5.0651 | 5.5250 | 5.6544 | 4.0692 | 4.0171 | 4.3552 |
| hsa_circ_0027141 | 0.0017 | 2.4324 | up | 6.8645 | 6.7680 | 7.1587 | 5.5985 | 5.8014 | 5.5618 |
| hsa_circ_0022870 | 0.0049 | 2.4341 | up | 5.2494 | 5.1980 | 5.4508 | 3.7220 | 4.0484 | 4.2431 |
| hsa_circ_0044561 | 0.0040 | 2.4362 | up | 5.2541 | 5.1363 | 5.2128 | 3.6947 | 4.0582 | 3.9742 |
| hsa_circ_0089680 | 0.0033 | 2.4512 | up | 6.0321 | 5.6132 | 5.6174 | 4.6135 | 4.3422 | 4.4550 |
| hsa_circ_0012485 | 0.0061 | 2.4616 | up | 5.8100 | 5.8887 | 5.7739 | 4.4500 | 4.7566 | 4.3362 |
| hsa_circ_0041564 | 0.0074 | 2.4864 | up | 4.2055 | 3.8532 | 3.5373 | 2.8858 | 2.3012 | 2.4809 |
| hsa_circ_0066769 | 0.0057 | 2.5880 | up | 5.5396 | 5.1732 | 5.2704 | 3.9710 | 3.9857 | 3.9359 |
| hsa_circ_0082169 | 0.0003 | 2.5893 | up | 5.6142 | 5.6054 | 5.6701 | 4.2748 | 4.3291 | 4.1641 |
| hsa_circ_0051916 | 0.0022 | 2.6017 | up | 4.6602 | 4.2294 | 4.3171 | 3.3077 | 2.8339 | 2.9181 |
| hsa_circ_0033759 | 0.0081 | 2.6028 | up | 4.6825 | 4.7295 | 5.0588 | 3.0162 | 3.6604 | 3.6006 |
| hsa_circ_0051088 | 0.0095 | 2.6041 | up | 4.8449 | 5.3239 | 5.4513 | 4.2510 | 3.5123 | 3.6744 |
| hsa_circ_0012073 | 0.0045 | 2.6239 | up | 5.2204 | 5.5674 | 5.6239 | 4.0880 | 4.1607 | 4.0164 |
| hsa_circ_0008759 | 0.0013 | 2.6423 | up | 6.6426 | 6.8370 | 7.0379 | 5.4518 | 5.5559 | 5.3221 |
| hsa_circ_0029162 | 0.0096 | 2.6501 | up | 5.4267 | 5.2684 | 5.2919 | 4.2209 | 3.8579 | 3.6337 |
| hsa_circ_0043211 | 0.0005 | 2.6524 | up | 5.0084 | 5.1814 | 5.3266 | 3.6640 | 3.8765 | 3.7636 |
| hsa_circ_0052543 | 0.0056 | 2.6962 | up | 5.7872 | 6.3154 | 6.0634 | 4.7082 | 4.6857 | 4.5203 |
| hsa_circ_0018665 | 0.0029 | 2.7062 | up | 6.8843 | 6.3793 | 6.4489 | 5.1954 | 5.2948 | 4.9449 |
| hsa_circ_0088667 | 0.0018 | 2.7071 | up | 4.0542 | 4.0251 | 3.7453 | 2.7560 | 2.4645 | 2.2719 |
| hsa_circ_0068611 | 0.0091 | 2.7118 | up | 4.6922 | 4.2586 | 4.1336 | 2.4700 | 3.2655 | 2.9831 |
| hsa_circ_0052322 | 0.0011 | 2.7185 | up | 4.8093 | 5.0520 | 4.7194 | 3.5477 | 3.5314 | 3.1619 |
| hsa_circ_0076550 | 0.0009 | 2.7259 | up | 7.3252 | 7.1635 | 7.5712 | 5.8218 | 6.0903 | 5.8200 |
| hsa_circ_0009847 | 0.0057 | 2.7521 | up | 5.1278 | 5.4598 | 5.5644 | 3.9988 | 3.9099 | 3.8947 |
| hsa_circ_0072552 | 0.0004 | 2.8001 | up | 4.3993 | 4.6632 | 4.7135 | 2.9932 | 3.2675 | 3.0638 |
| hsa_circ_0010074 | 0.0083 | 2.8392 | up | 7.1553 | 7.4925 | 7.8086 | 5.9541 | 6.1462 | 5.9014 |
| hsa_circ_0041199 | 0.0007 | 2.8429 | up | 5.4556 | 5.2724 | 5.1140 | 4.0027 | 3.6293 | 3.6789 |
| hsa_circ_0077076 | 0.0031 | 2.8800 | up | 6.4412 | 6.1627 | 5.9100 | 4.7719 | 4.8605 | 4.2896 |
| hsa_circ_0078930 | 0.0034 | 2.8839 | up | 5.7470 | 5.3565 | 5.5726 | 4.0996 | 4.2758 | 3.6800 |
| hsa_circ_0049294 | 0.0053 | 2.8997 | up | 4.5133 | 5.0502 | 5.1353 | 3.1564 | 3.5352 | 3.4481 |
| hsa_circ_0055856 | 0.0001 | 2.9160 | up | 7.8903 | 7.7510 | 7.9006 | 6.3332 | 6.4013 | 6.1705 |
| hsa_circ_0025259 | 0.0056 | 2.9977 | up | 6.9684 | 6.4549 | 6.3720 | 4.9962 | 5.2037 | 4.9020 |
| hsa_circ_0009402 | 0.0030 | 3.0244 | up | 4.9699 | 4.9369 | 5.0499 | 3.2421 | 3.2864 | 3.6118 |
| hsa_circ_0075648 | 0.0008 | 3.0409 | up | 5.8727 | 5.6059 | 5.5474 | 4.3226 | 3.8983 | 3.9768 |
| hsa_circ_0073642 | 0.0090 | 3.0991 | up | 5.1712 | 5.6752 | 5.6304 | 3.4452 | 3.7239 | 4.3215 |
| hsa_circ_0033725 | 0.0054 | 3.0994 | up | 5.7079 | 6.1855 | 6.1098 | 4.6242 | 4.5175 | 3.9130 |
| hsa_circ_0083829 | 0.0011 | 3.1057 | up | 6.5723 | 6.5703 | 6.8424 | 5.0348 | 5.0894 | 4.9710 |
| hsa_circ_0083828 | 0.0034 | 3.1315 | up | 5.8925 | 6.2144 | 6.5716 | 4.8432 | 4.6442 | 4.2740 |
| hsa_circ_0040879 | 0.0077 | 3.1533 | up | 4.7368 | 5.2356 | 5.3520 | 3.5045 | 3.5622 | 3.3497 |
| hsa_circ_0056415 | 0.0044 | 3.2124 | up | 4.4954 | 4.2968 | 3.9734 | 2.5842 | 2.1659 | 2.9155 |
| hsa_circ_0012123 | 0.0042 | 3.2196 | up | 5.9014 | 5.8466 | 5.7891 | 4.0561 | 4.4091 | 3.9758 |
| hsa_circ_0067211 | 0.0031 | 3.2282 | up | 6.3741 | 6.7103 | 6.7231 | 5.2082 | 4.5610 | 4.9206 |
| hsa_circ_0075410 | 0.0041 | 3.2508 | up | 6.7760 | 6.8699 | 6.7297 | 5.0325 | 5.3434 | 4.8586 |
| hsa_circ_0084203 | 0.0004 | 3.3355 | up | 4.7114 | 4.8912 | 4.4877 | 2.9262 | 3.1372 | 2.8235 |
| hsa_circ_0055857 | 0.0005 | 3.3430 | up | 7.7307 | 7.6525 | 7.8703 | 6.1948 | 6.0002 | 5.8190 |
| hsa_circ_0060168 | 0.0011 | 3.3949 | up | 4.2586 | 4.1881 | 3.7661 | 2.5838 | 2.1908 | 2.1553 |
| hsa_circ_0046232 | 0.0005 | 3.4815 | up | 5.5263 | 5.4658 | 5.8710 | 3.9047 | 3.9828 | 3.5795 |
| hsa_circ_0069020 | 0.0017 | 3.5479 | up | 6.5115 | 6.4437 | 6.1506 | 4.7712 | 4.6198 | 4.2001 |
| hsa_circ_0060381 | 0.0027 | 3.5613 | up | 6.1200 | 5.9425 | 5.5656 | 3.9832 | 4.2089 | 3.9809 |
| hsa_circ_0012122 | 0.0012 | 3.6058 | up | 6.4337 | 6.5750 | 6.4395 | 4.6983 | 4.7735 | 4.4042 |
| hsa_circ_0090581 | 0.0045 | 3.6749 | up | 4.8987 | 5.7138 | 5.4796 | 3.7289 | 3.6736 | 3.0911 |
| hsa_circ_0013663 | 0.0003 | 3.6750 | up | 4.5846 | 4.6793 | 4.3488 | 2.6907 | 2.8446 | 2.4346 |
| hsa_circ_0025832 | 0.0001 | 3.6778 | up | 5.3125 | 5.5613 | 5.3375 | 3.6555 | 3.5509 | 3.3670 |
| hsa_circ_0022865 | 0.0007 | 3.7128 | up | 6.2315 | 6.1673 | 6.1552 | 4.1550 | 4.4175 | 4.2932 |
| hsa_circ_0068188 | 0.0001 | 3.8292 | up | 4.8915 | 5.0987 | 4.9788 | 3.1416 | 3.1207 | 2.8900 |
| hsa_circ_0069029 | 0.0036 | 3.8488 | up | 5.2296 | 5.5472 | 5.3138 | 3.6923 | 3.4863 | 3.0177 |
| hsa_circ_0000186 | 0.0007 | 3.8807 | up | 6.0560 | 5.9771 | 5.6812 | 4.0026 | 4.0204 | 3.8422 |
| hsa_circ_0082816 | 0.0002 | 3.9854 | up | 7.0410 | 6.9879 | 6.8301 | 4.8444 | 5.1441 | 4.8755 |
| hsa_circ_0073641 | 0.0024 | 4.1840 | up | 5.0504 | 5.7557 | 5.5762 | 3.3794 | 3.1951 | 3.6635 |
| hsa_circ_0072273 | 0.0016 | 4.3675 | up | 5.0325 | 5.4214 | 5.1501 | 3.0762 | 3.3808 | 2.7194 |
| hsa_circ_0072275 | 0.0098 | 4.3877 | up | 4.2089 | 4.8928 | 4.4942 | 2.8753 | 2.4647 | 1.7070 |
| hsa_circ_0017587 | 0.0044 | 4.3982 | up | 4.8560 | 4.3996 | 4.3050 | 2.8555 | 2.2700 | 1.9326 |
| hsa_circ_0002919 | 0.0070 | 4.4197 | up | 4.4366 | 5.2098 | 4.5288 | 2.5848 | 3.0952 | 1.9823 |
| hsa_circ_0055789 | 0.0043 | 4.4351 | up | 5.8206 | 6.2901 | 6.5178 | 4.1724 | 4.1550 | 3.9262 |
| hsa_circ_0013428 | 0.0040 | 4.5423 | up | 6.1948 | 6.8690 | 6.8012 | 4.3665 | 4.6459 | 4.3750 |
| hsa_circ_0048133 | 0.0058 | 4.6295 | up | 3.9731 | 3.8132 | 3.7181 | 2.0133 | 1.2343 | 1.5269 |
| hsa_circ_0055625 | 0.0014 | 4.6879 | up | 6.0209 | 6.5973 | 6.6019 | 4.3150 | 4.4355 | 3.7742 |
| hsa_circ_0063330 | 0.0021 | 4.8026 | up | 5.8641 | 6.5385 | 6.3502 | 3.8386 | 4.2001 | 3.9805 |
| hsa_circ_0008486 | 0.0004 | 4.9809 | up | 5.1718 | 5.3962 | 5.1414 | 2.9799 | 2.9012 | 2.8912 |
| hsa_circ_0013427 | 0.0012 | 5.2486 | up | 6.2901 | 6.9137 | 6.9552 | 4.4431 | 4.5648 | 4.0092 |
| hsa_circ_0009539 | 0.0028 | 5.3310 | up | 5.0419 | 5.8874 | 5.4737 | 2.9494 | 2.9359 | 3.3564 |
| hsa_circ_0015646 | 0.0075 | 5.8902 | up | 5.4197 | 5.7745 | 5.6707 | 3.3702 | 3.2546 | 2.4126 |
| hsa_circ_0043874 | 0.0034 | 5.9630 | up | 5.3598 | 6.2438 | 6.2070 | 3.7305 | 3.3197 | 3.1252 |
| hsa_circ_0067417 | 0.0048 | 6.3251 | up | 5.0356 | 5.9734 | 5.6262 | 3.2302 | 3.1903 | 2.1427 |
| hsa_circ_0063332 | 0.0029 | 6.3506 | up | 5.6788 | 6.3733 | 6.4083 | 3.5182 | 3.6954 | 3.3337 |
| hsa_circ_0000375 | 0.0004 | 6.7287 | up | 6.3190 | 6.6792 | 6.8415 | 3.8491 | 4.1774 | 3.5403 |
| hsa_circ_0063331 | 0.0028 | 7.0699 | up | 5.1383 | 6.1977 | 5.8536 | 2.7458 | 3.3739 | 2.6934 |
| hsa_circ_0009135 | 0.0008 | 9.0104 | up | 4.0240 | 4.8404 | 4.5348 | 1.5102 | 1.5747 | 0.7909 |

Supplementary Table 4 miRNAs that might potentially bind to circNHPH4

| Mirbase ID | Score percentile |
| --- | --- |
| hsa-miR-1205 | 90 |
| hsa-miR-1231 | 92 |
| hsa-miR-136 | 82 |
| hsa-miR-140-3p | 85 |
| hsa-miR-217 | 75 |
| hsa-miR-330-5p | 72 |
| hsa-miR-515-5p | 80 |
| hsa-miR-516b | 81 |
| hsa-miR-556-5p | 73 |
| hsa-miR-558 | 83 |

**Supplementary Table 5 The demographic characteristics and CAD-relevant indicators in CAD patients and control participants**

| Variables | CAD patients  n=81 | Control subjects  n=77 | p-value |
| --- | --- | --- | --- |
| Age (years) | 63.84±10.10 | 63.36±5.32 | 0.717 |
| Male | 29（28.16%） | 27（40.30%） | 0.100 |
| BMI(kg/m2) | 24.63±3.98 | 25.17±3.30 | 0.360 |
| HBP（%） | 71(68.93%) | 35(52.24%) | 0.028^**^ |
| T_2_DM（%） | 35(33.98%) | 5(7.46%) | ＜0.001^**^ |
| Smoking（%） | 52(50.49%) | 30(44.78%) | 0.467 |
| Drinking（%） | 23(22.33%) | 18(26.87%) | 0.499 |
| MAP(mmHg) | 92.29±11.13 | 92.10±10.97 | 0.913 |
| HR（bpm） | 74.29±9.67 | 74.28±11.64 | 0.996 |
| TG (mmol/L) | 1.74±1.20 | 1.57±0.73 | 0.276 |
| TC (mmol/L) | 4.24±1.16 | 4.37±1.09 | 0.443 |
| HDL-C (mmol/L) | 1.18±0.25 | 1.29±0.31 | 0.009^**^ |
| LDL-C (mmol/L) | 2.53±0.83 | 2.67±0.78 | 0.296 |
| Apo-a(g/l) | 1.31±0.27 | 1.37±0.35 | 0.188 |
| Apo-b(g/l) | 0.79±0.27 | 0.80±0.24 | 0.873 |
| Lpa(mg/l) | 308.59±406.17 | 194.43±277.75 | 0.046^**^ |
| Uric acid (umol/l) | 382.06±106.72 | 352.08±104.09 | 0.072 |
| FPG(mmol/L) | 6.14±2.24 | 5.59±0.74 | 0.054 |

BMI, body mass index; HBP, high blood pressure; T_2_DM, type 2 diabetes; MAP, mean arterial pressure；HR, heart rate; TG, triglyceride; TC, total cholesterol; HDL-C, high density lipoprotein; LDL-C, low density lipoprotein; Apo-a, apolipoprotein-a; Apo-b, apolipoprotein-b; Lpa, lipoprotein a; FPG, fasting plasma glucose.

**Supplementary Table 6** **Correlation between baseline characteristic and circRNAs level in CAD patients**

|  | Exosomal circNPHP4 | | Plasma circNPHP4 | |
| --- | --- | --- | --- | --- |
|  | p-value | Coefficient | p-value | Coefficient |
| Age (years) | 0.215 | -0.142 | 0.306 | -0.117 |
| Male | 0.490 | 0.069 | 0.127 | -0.151 |
| BMI(kg/m2) | 0.047^*^ | -0.223 | 0.256 | -0.128 |
| HBP（%） | 0.043^*^ | -0.199 | 0.200 | -0.127 |
| T_2_DM（%） | 0.589 | 0.054 | 0.036^*^ | -0.207 |
| Smoking（%） | 0.034^*^ | 0.156 | 0.036^*^ | 0.128 |
| Drinking（%） | 0.012^*^ | 0.058 | 0.000^**^ | 0.211 |
| MAP(mmHg) | 0.566 | -0.065 | 0.772 | 0.033 |
| HR（bpm） | 0.508 | 0.074 | 0.650 | -0.053 |
| TG (mmol/L) | 0.813 | 0.027 | 0.704 | -0.043 |
| TC (mmol/L) | 0.044^*^ | 0.226 | 0.288 | 0.120 |
| HDL-C (mmol/L) | 0.569 | -0.065 | 0.606 | -0.059 |
| LDL-C (mmol/L) | 0.018^*^ | 0.265 | 0.164 | 0.157 |
| Apo-a(g/l) | 0.279 | -0.123 | 0.286 | -0.121 |
| Apo-b(g/l) | 0.021^*^ | 0.258 | 0.162 | 0.158 |
| Lpa(mg/l) | 0.996 | 0.001 | 0.928 | 0.010 |
| Uric acid (umol/l) | 0.062 | 0.209 | 0.195 | 0.146 |
| FPG(mmol/L) | 0.912 | 0.012 | 0.514 | -0.074 |
| Syntax score | 0.006^**^ | 0.303 | 0.017^*^ | 0.263 |

BMI, body mass index; HBP, high blood pressure; T2DM, type 2 diabetes; MAP, mean arterial pressure；HR, heart rate; TG, triglyceride; TC, total cholesterol; HDL-C, high density lipoprotein; LDL-C, low density lipoprotein; Apo-a, apolipoprotein-a; Apo-b, apolipoprotein-b; Lpa, lipoprotein a; FPG, fasting plasma glucose.

**Supplementary codes**

#forest plot

rt <- read.table("mul.txt",header=T,sep="\t",row.names=1,check.names=F)

gene <- rownames(rt)

hr <- sprintf("%.3f",rt$"HR")

hrLow <- sprintf("%.3f",rt$"HR.95L")

hrHigh <- sprintf("%.3f",rt$"HR.95H")

Hazard.ratio <- paste0(hr,"(",hrLow,"-",hrHigh,")")

pVal <- ifelse(rt$pvalue<0.001, "<0.001", sprintf("%.3f", rt$pvalue))

pdf(file="forest-unicox.pdf", width = 10,height =12)

n <- nrow(rt)

nRow <- n+1

ylim <- c(1,nRow)

layout(matrix(c(1,2),nc=2),width=c(3,2))

xlim = c(0,3)

par(mar=c(4,2.5,2,1))

plot(1,xlim=xlim,ylim=ylim,type="n",axes=F,xlab="",ylab="")

text.cex=0.8

text(0,n:1,gene,adj=0,cex=text.cex)

text(1.5-0.5*0.2,n:1,pVal,adj=1,cex=text.cex);text(1.5-0.5*0.2,n+1,'pvalue',cex=text.cex,font=2,adj=1)

text(3,n:1,Hazard.ratio,adj=1,cex=text.cex);text(3,n+1,'Hazard ratio',cex=text.cex,font=2,adj=1,)

par(mar=c(4,1,2,1),mgp=c(2,0.5,0))

xlim = c(0,max(as.numeric(hrLow),as.numeric(hrHigh)))

plot(1,xlim=xlim,ylim=ylim,type="n",axes=F,ylab="",xaxs="i",xlab="Hazard ratio")

arrows(as.numeric(hrLow),n:1,as.numeric(hrHigh),n:1,angle=90,code=3,length=0.05,col="darkblue",lwd=2.5)

abline(v=1,col="black",lty=2,lwd=2)

boxcolor = ifelse(as.numeric(hr) > 1, 'red', 'green')

points(as.numeric(hr), n:1, pch = 15, col = boxcolor, cex=1.3)

axis(1)

dev.off()

library(glmnet)

v1<-as.matrix(mydata[,c(3:38)])

v2 <-mydata[,2]

myfit <- glmnet(v1, v2, family = "binomial")

pdf("lambda2.pdf")

plot(myfit, xvar = "lambda", label = TRUE)

dev.off()

myfit2 <- cv.glmnet(v1, v2, family="binomial")

pdf("min2.pdf")

plot(myfit2)

abline(v=log(c(myfit2$lambda.min,myfit2$lambda.1se)),lty="dashed")

dev.off()

myfit2$lambda.min

coe <- coef(myfit, s = myfit2$lambda.min)

act_index <- which(coe != 0)

act_coe <- coe[act_index]

row.names(coe)[act_index]

#nomogram

library(rms)

non_tumor<-read.table("clinical1.txt",header=T,sep="\t")

non_tumor$sex<-factor(non_tumor$sex,labels=c("female","male"))

non_tumor$alcohol<-factor(non_tumor$alcohol,labels=c("no","yes"))

non_tumor$age<-factor(non_tumor$age,labels=c("＜63","≥63"))

non_tumor$BP<-factor(non_tumor$BP,labels=c("normal","hypertenssion"))

non_tumor$apoa<-factor(non_tumor$apoa,labels=c("normal","lower"))

non_tumor$Lpa<-factor(non_tumor$Lpa,labels=c("＜140","≥140"))

non_tumor$Circexosomal<-factor(non_tumor$Circexosomal,labels=c("＜4.679","≥4.679"))

non_tumor$Circplasma<-factor(non_tumor$Circplasma,labels=c("＜2.9","≥2.9"))

non_tumor$T2DM<-factor(non_tumor$T2DM,labels=c("NO","YES"))

ddist <- datadist(non_tumor)

options(datadist="ddist")

mylog<- glm(status~ sex + alcohol + age + BP + apoa + Lpa + Circexosomal + Circplasma + T2DM,family=binomial(link = "logit"),data = non_tumor)

summary(mylog)

coefficients(mylog)

exp(coefficients(mylog))

exp(confint(mylog))

mylog<-lrm(status~sex + alcohol + BP + Lpa + Circexosomal + Circplasma + T2DM,data=non_tumor,x=T,y=T)

mynom<- nomogram(mylog, fun=plogis,fun.at=c(0.0001,0.1,0.2,0.3,0.4,0.5,0.6,0.7,0.8,0.9,0.9999),lp=F, funlabel="risk of CAD")

pdf("Nom.pdf",10,8)

plot(mynom)

dev.off()

#c-index

mylog<-lrm(status~sex + alcohol + age + BP + apoa + Lpa + Circexosomal + Circplasma + T2DM,data=non_tumor,x=T,y=T)

set.seed(300)

myc<-validate(mylog,method="b",B = 1000,pr=T,dxy=T)

c_index<-(myc[1,8]+1)/2

c_index

#calibrate

mylog<-lrm(status~sex + alcohol + age + BP + apoa + Lpa + Circexosomal + Circplasma + T2DM,data=non_tumor,x=T,y=T)

mycal<-calibrate(mylog,method="boot",B=1000)

pdf("Calibration-dev.pdf")

plot(mycal,xlab="Nomogram-predicted probability of non-remission",ylab="Actual diagnosed non-remission (proportion)",sub=T)

dev.off()

#DCA

library(rms)

library(rmda)

non_tumor<-read.table("clinical.txt",header=T,sep="\t")

modul1<- decision_curve(status~ sex + alcohol + age + BP + apoa + Lpa + Circexosomal + Circplasma + T2DM

,data= non_tumor,

family = binomial(link ='logit'),

thresholds= seq(0,1, by = 0.01),

confidence.intervals = 0.95)

modul2<- decision_curve(status~ sex + alcohol + age + BP + apoa + Lpa + Circplasma + T2DM

,data= non_tumor,

family = binomial(link ='logit'),

thresholds= seq(0,1, by = 0.01),

confidence.intervals = 0.95)

modul3<- decision_curve(status~ sex + alcohol + age + BP + apoa + Lpa + Circexosomal + T2DM

,data= non_tumor,

family = binomial(link ='logit'),

thresholds= seq(0,1, by = 0.01),

confidence.intervals = 0.95)

modul4<- decision_curve(status~ sex + alcohol + age + BP + apoa + Lpa + T2DM

,data= non_tumor,

family = binomial(link ='logit'),

thresholds= seq(0,1, by = 0.01),

confidence.intervals = 0.95)

pdf("DCA.pdf")

plot_decision_curve(list(modul1,modul2,modul3,modul4),

curve.names= c("Circexosomal and Circplasma nomogram","model disintegrating Circexosomal", "model disintegrating Circplasma", "model disintegrating Circexosomal and Circplasma"), xlab="Threshold probability",

cost.benefit.axis =FALSE,col=c( "blue","red","purple","green"),

confidence.intervals=FALSE,

standardize = FALSE)

dev.off()
